# Game On? Smoking Cessation Through the Gamification of mHealth: A Longitudinal Qualitative Study

**DOI:** 10.2196/games.5678

**Published:** 2016-10-24

**Authors:** Abdulrahman Abdulla El-Hilly, Sheeraz Syed Iqbal, Maroof Ahmed, Yusuf Sherwani, Mohammed Muntasir, Sarim Siddiqui, Zaid Al-Fagih, Omar Usmani, Andreas B Eisingerich

**Affiliations:** ^1^ Faculty of Medicine Department of Medicine Imperial College London London United Kingdom; ^2^ Airway Disease National Heart and Lung Institute Imperial College London & Royal Brompton Hospital London United Kingdom; ^3^ Imperial College Business School Imperial College London London United Kingdom

**Keywords:** gamification, mhealth, mobile health, smoking cessation, health behavior, health policy, public health, behavioral support

## Abstract

**Background:**

Finding ways to increase and sustain engagement with mHealth interventions has become a challenge during application development. While gamification shows promise and has proven effective in many fields, critical questions remain concerning how to use gamification to modify health behavior.

**Objective:**

The objective of this study is to investigate how the gamification of mHealth interventions leads to a change in health behavior, specifically with respect to smoking cessation.

**Methods:**

We conducted a qualitative longitudinal study using a sample of 16 smokers divided into 2 cohorts (one used a gamified intervention and the other used a nongamified intervention). Each participant underwent 4 semistructured interviews over a period of 5 weeks. Semistructured interviews were also conducted with 4 experts in gamification, mHealth, and smoking cessation. Interviews were transcribed verbatim and thematic analysis undertaken.

**Results:**

Results indicated perceived behavioral control and intrinsic motivation acted as positive drivers to game engagement and consequently positive health behavior. Importantly, external social influences exerted a negative effect. We identified 3 critical factors, whose presence was necessary for game engagement: purpose (explicit purpose known by the user), user alignment (congruency of game and user objectives), and functional utility (a well-designed game). We summarize these findings in a framework to guide the future development of gamified mHealth interventions.

**Conclusions:**

Gamification holds the potential for a low-cost, highly effective mHealth solution that may replace or supplement the behavioral support component found in current smoking cessation programs. The framework reported here has been built on evidence specific to smoking cessation, however it can be adapted to health interventions in other disease categories. Future research is required to evaluate the generalizability and effectiveness of the framework, directly against current behavioral support therapy interventions in smoking cessation and beyond.

## Introduction

Smoking is responsible for 19% of all deaths in the United Kingdom, with a direct cost of £5.2 billion to the National Health Service (NHS) [1]. It is a leading cause of chronic disease [2], and has been declared as the most important cause of preventable morbidity and premature mortality worldwide [3]. However, depressingly, there remains a significant disparity between individuals desiring to quit smoking (~68%), and those actually successfully quitting (~3%) [4]. In 2013/14, the NHS Stop Smoking Services reached only 9% of individuals in the United Kingdom seeking to quit. Alarmingly, this represented a 19% year-on-year reduction [5,6]. As a result, the NHS 5-year forward view pledged ‘hard-hitting national action’ against preventable diseases including smoking, with a new set of smoking cessation services being outlined by Public Health England to promote healthier behavior [7].

A Cochrane review concluded that high-intensity behavioral support combined with pharmacological intervention was the most effective method for smoking cessation [8] with the National Institute for Health and Care Excellence demonstrating a 35% quit rate compared with a 2% background quit rate. However, this approach is expensive with lifetime costs of £7010 per person, where behavioral support contributes to the bulk of the cost [9]. The continuing economic and societal burden created by smoking suggests current smoking cessation techniques are underserving the population and begs the question: are there any other novel approaches we can take to tackle the addiction of smoking?

The rapid technological advancement of mobile phone technologies over the last decade has facilitated a burgeoning market for mHealth apps. However, while thousands of mHealth apps have been released, most have fallen short of their grand expectations owing largely to poor user engagement levels [10]. User engagement was identified as a critical factor to the success of mHealth in an analysis of 945 mHealth apps [11]. Thus, finding ways to increase user engagement with their target audience has become a significant focus of mHealth interventions [12].

Gamification is ‘the use of game design elements in nongame contexts’ [13], making use of the potential motivational ability of games. Gamification empowers users to complete tasks more efficiently, while making them more enjoyable, with the aim of increasing engagement [14]. Cugelman [15] argues that gamification is only effective when used in conjunction with academically grounded behavioral change strategies, and goes on to identify 7 “core ingredients” that can be used as persuasive strategies to promote behavioral change.

The application of gamification in mHealth is an emerging field. *Sparx*, a digital game intervention developed to treat clinical depression in adolescents, represents a successful implementation of gamification. A randomized controlled trial demonstrated noninferiority of the game against traditional face-to-face counseling, along with significantly higher remission rates [16]. From the perspective of health behavior, gamification has shown promising results in encouraging physical activity by turning the ‘work of exercise’ into a game [17]. A recent review published in JMIR Mental Health found no studies had been published explicitly examining the role of gamification features on program adherence with Web-based interventions to manage common mental health disorders [18].

**Figure 1:**
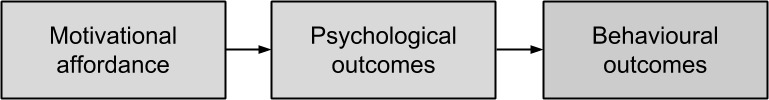
How motivational affordances lead to behavioral outcomes [19].

Many health apps have attempted to replicate such success by promoting positive health behavior in a wider context, particularly in relation to smoking, albeit with variable success [12]. A systematic literature review found that the implementation of game elements helps to create motivational affordances that lead to desired psychological outcomes and the consequent behavioral outcome (Figure 1) [19]. However, critical questions remain concerning the mechanism by which gamification exerts its influence, with a particular paucity in research surrounding gamification in the context of health behavior.

While gamification shows promise and has proven effective in many fields, research is required to investigate the beneficial effects on health behavior and disease self-management to warrant the implementation of such interventions [20]. The aim of this study was to take a cognitivist approach, building on the evidence gathered from existing literature and our own data collection, to gain insight into the underlying thought processes and internal rules, which govern the way individuals react to a gamified smoking cessation intervention. Consequently, in this exploratory work, we aim to suggest how gamification might lead to a change in health behavior specifically with respect to smoking cessation.

**Table 1:** Intervention comparison.

Game Components	Kwit 2	Puff Away
Store rating	Apple store - 4.5 **/**5	Google Play - 4.2 /5
Goal setting	Progress tracking Level system	Nonexistent
Capacity to overcome challenges	Information facilitating growth Learning and development	Nonexistent
Providing feedback on performance	Messages via achievement system	Nonexistent
Reinforcement	Rewards from levels and achievements Avoiding punishment associated with smoking	Nonexistent
Comparing progress	Nonexistent	Nonexistent
Social connectivity	Facebook Twitter	Nonexistent
Fun and playfulness	Minimal	Nonexistent

## Methods

### Study Approach

We conducted a qualitative longitudinal study with 16 smokers in 2 cohorts. The first cohort used a nongamified mHealth intervention free of any game components, while the second used a gamified mHealth intervention.

### Interventions

In order to isolate the game-specific effects we would require 2 identical apps, differing only by the presence of gamified features. To approach this level of distinction, we shortlisted, downloaded, and tested 12 apps to establish which, gamification aside, were the most similar in app mechanics to allow fair comparison. ‘Puff Away’ and ‘Kwit 2’ were chosen because they both used very similar mechanisms to engage the user, focussing on tackling user education and providing progress tracking. The additional game components in Kwit 2 are specified in Table 1 [15].

### Participants

All participants met the following 4 criteria: a smoker currently intent on quitting; 18+ years old; English speaker; and owner of a smartphone. We excluded those with smoking-related illnesses. Participants were recruited from local smoking support groups and university campuses in London. Each participant was then randomly allocated to a cohort and asked to install the relevant app onto their own smartphones. Participants were not compensated for their time. Informed verbal and written consent was obtained prior to commencing the study. The study had approval from the Imperial College Research Ethics Committee.

### Interview Procedure

We conducted semistructured, one-on-one interviews with participants (30 minutes). Four interviews were conducted with each participant over the course of 5 weeks. The first interview (week 0) assessed their smoking background and demographics. This was accompanied by a standardized set of instructions on how to use their specific app. Subsequent interviews were then conducted at weeks 1, 3, and 5, with changes in participant behavior and emotions being tracked and recorded. Interviewers were instructed to neither encourage nor discourage the participant’s smoking behavior so as to minimize any effect on their behavior. Interview questions were formulated and then discussed with 2 independent, experienced qualitative researchers. The participants were asked about their progress in relation to smoking cessation, their experience using the app, the effect of the app on their behavior and emotions, as well as the specific effects of the game components. The final interview incorporated an exit interview in which participants expressed their overall experience. We conducted an internal pilot to test our methodology with 5 participants. Week 0 and week 1 interviews were conducted with each participant, allowing us to refine the interview questions and confirm the suitability of the 2 apps selected. The methodology employed with the pilot study was deemed satisfactory for the main study and so the results of all 5 pilot participants were included in the full longitudinal study. A semistructured interview guide can be found in the Web-based supplement in Multimedia Appendix 1.

### Analysis Procedure

The 6-phase analytic framework was employed in our thematic analysis [21]. Audiotaped interviews were transcribed and read by 3 researchers on 2 separate occasions. These interview scripts were then used to manually generate codes for recurring patterns across participants. The codes were then analyzed to form overarching themes, all of which were defined by the 3 researchers. Ambiguities were resolved in discussion. This was a recursive process, whereby researchers cycled back and forth through the phases to allow for iteration as required. Once the saturation point had been reached and no new themes were emerging, the recruitment of new participants was stopped, conforming to the grounded theory approach [22]. All data regarding theme construction and interpretation was recorded in a reflexivity journal. Once the themes had been completed and defined, the researchers went back to the initial data sample to verify the accuracy of the overarching themes.

### Expert Interviews

Semistructured interviews with 4 experts were conducted. The experts were initially shortlisted following our literature search, subsequently looking specifically at the research credentials of candidates. This shortlist was then narrowed to 4 based on their level of expertise within their respective fields of gamification, digital health, and smoking cessation. The experts were: Prof Scott Nicholson, Professor of Game Design and Development, and Director at Because Play Matters game lab, Wilfrid Laurier University; Prof Steven Johnson, Assistant Professor at Fox School of Business, Temple University; Dr Dominic King, Coauthor of the most cited editorial on Gamification and Health Behavior Change; and Dr Omar Usmani, Reader in Respiratory Medicine and Consultant Physician in Respiratory Medicine at the National Heart and Lung Institute, Imperial College London & Royal Brompton Hospital. A semistructured interview was conducted with each expert affording an in-depth multifaceted exploration of both gamification and smoking cessation. The transcripts from these interviews underwent the same manual thematic coding procedure as outlined for the longitudinal participant interviews.

## Results

### Participant Characteristics

Of the 19 participants initially recruited, 3 participants dropped out after the week 0 interview. Of the 3 dropouts, 2 were in the gamified cohort and 1 was in the nongamified cohort. The reasons given for dropout were refusal of further contact (2) and problems with availability (1). The resulting analysis is of the remaining 16 participants: 9 used the gamified intervention and 7 used the nongamified intervention. All 16 participants reported daily use of their smartphones. All participants expressed a desire to quit smoking prior to recruitment to the study, with 31% (5/16) of the participants attempting to quit for the first time. Additional characteristics are summarized in Table 2

**Table 2:** Participant characteristics.

Characteristic		Gamified Users (n=9)	Non-Gamified users (n=7)
		n (%)	n (%)
**Mean age**		26.22 (range, 18-45)	28.1 (range, 20-52)
**Cultural split**			
	South Asian	5 (56)	2 (29)
	Arab	2 (22)	1 (14)
	Caucasian/British	2 (22)	2 (29)
	East Asian	-	2 (29)
**Gender**			
	Female	3 (33)	2 (29)
	Male	6 (67)	5 (71)
**Occupation**			
	Student (undergraduate and postgraduate)	6 (67)	6 (86)
	Working(part-time or full-time)	4 (44)	2 (29)

**Figure 2:**
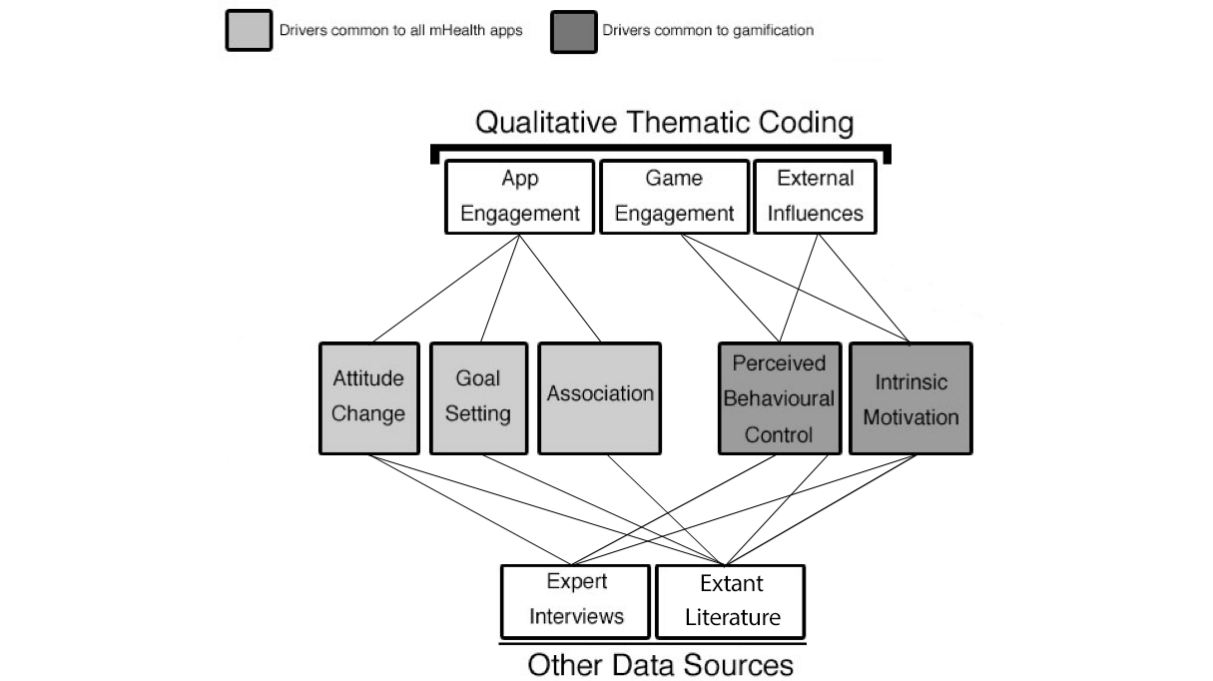
Analysis of results.

**Figure 3:**
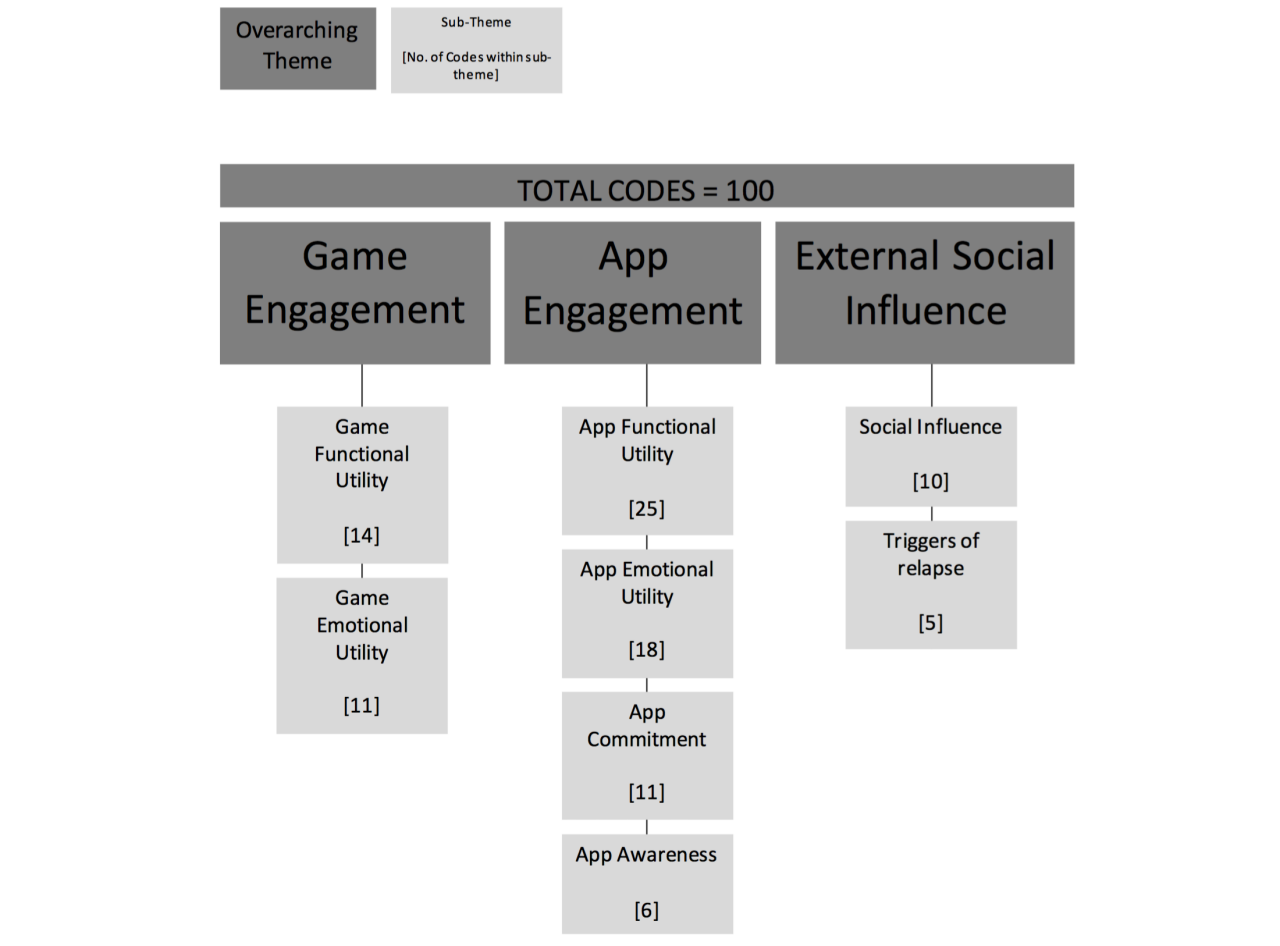
Thematic analysis.

### Longitudinal Participant Interviews

A total of 57 interviews were conducted across a 5-week period: 16 interviews at week 0, 16 at week 1, 11 at week 3, and 14 at week 5. Transcripts and audio recordings were available for all the interviews.

Three overarching themes that influenced the impact of the app intervention on health behavior were identified from the longitudinal participant interviews only, with 8 subthemes (Figure 2): app engagement, game engagement, and external influences. App engagement refers to the components common to both gamified and nongamified interventions that helped to create engagement with the user. Game engagement refers to those game components unique to the gamified intervention that helped build engagement with the user. Finally, external influence describes the factors external to both interventions that impacted engagement with the app. The number of codes found in each subtheme can be found in Figure 3.

### Expert Interviews

There was a consensus among the experts that technology has the potential to support health care professionals, in providing the behavioral support necessary in certain segments of the population. However, the experts questioned the long-term impact of a gamified intervention, and stated it would be a challenge to maintain long-term user commitment. Experts suggested that gamification can only reinforce desired behaviors, interventions should aim to build intrinsic motivation, rewards should be variable, and that a self-relevant experience is a critical success factor to building engagement with the game.

### Analysis of Results

Following a thorough analysis of our findings, we identified drivers and modifiers to health behavior change. Drivers describe the key mechanisms by which behavioral change is produced. Modifiers were identified as those factors whose presence and quality determined the strength of the drivers, and therefore how likely the app was to promote positive health behavior.

We recognized 3 drivers common to the mHealth interventions: attitude change, goal setting, and association (of the mHealth solution with the maladaptive health behavior). Additionally, 2 drivers were proposed as the method by which gamification promotes positive health behavior: perceived behavioral control and intrinsic motivation. Figure 2 illustrates the relationship between the themes from the participant interviews, the other data sources and the drivers to behavioral change.

### Drivers and Modifiers Common to the mHealth Interventions

#### Driver 1 - Attitude Change

A change in attitude toward the maladaptive health behavior described by 1 participant in the following statement:

It makes me reconsider if I really need to smoke or not and sometimes that extra few seconds is enough to put my cigarette away… It makes you contemplate and double think ‘Do I really need a cigarette right now?’

This was seen repeatedly with another participant explaining the following:

When someone is trying to quit it’s like a battle in your head [between smoking and not smoking] … the app helps you in this battle [to not smoke].

This finding can be explained by the Health Belief Model, which states that positive behavioral change can arise from increasing perceived threat of the negative health behavior and increasing perceived benefit of the positive health behavior [23]. Dr Usmani, with a background in smoking cessation, reinforced this by explaining how highlighting the implications of an individual’s actions may result in behavior change:

First of all contextualizing the advantage of stopping smoking gives them a scare, a bit of a shock… this is what happens in real life terms of making people want to quit smoking.

#### Driver 2 - Goal Setting

The progress tracking mechanism provided a simple visual means for participants to keep track of their efforts with participants appreciating the importance of such features:

It gives [me] nice visuals. Sometimes it’s hard to visualize exactly what a cigarette means but the bars help you visualize it.

Others felt the impact even more stating that:

it does help [motivate me] … especially with willpower.

Participants also reported a sense of commitment and duty toward the goals of the app:

It feels like I’ve committed to this, so I am more motivated to try and make it work.

With some this commitment was often expressed through some sense of guilt when they smoked:

When I smoked it’s like cheating… you betray yourself when you press the button… Now when I think about it I feel horrendously guilty [when I smoked].

This commitment was more common among users of the gamified intervention with only 1 participant expressing commitment in the nongamified cohort, but 4 expressing this feeling in the gamified cohort in week 1 interviews. PRIME theory states that in the context of smoking cessation, an intention or commitment is required before an individual can be motivated to change their behavior [24].

#### Driver 3 - Association

Participants began to associate the act of smoking with the mHealth interventions with a participant explicitly voicing this:

The app helped me create an associated between app and smoking.

Another user went further to explain the change in his habit with the following:

My routine has changed now, when I get my cigarette out I automatically get my phone out as well now. The app has become integrated into my smoking.

**The five modifiers of mHealth interventions.**

Extant knowledge: the usefulness of information provided was inversely proportional to the user’s existing knowledge. User’s persistently commented on meaninglessness of repeated information:

*All this information is out there already; I need something with more information [that is not known by me].*

Ease of use: perceived simplicity increased engagement. The less convenient and simple the app was to use the lower the level of engagement of the user:

*[It’s] Impractical, when you reach for a cigarette the last thing you’re thinking about is pulling out your phone and update the app… you’ve made your decision and you’re over that dilemma.*

Aesthetics: an attractive app design increased engagement with participants specifically mentioning unenticing visuals:

*[It is] difficult to engage in the app due to poor presentation and poor visuals.*

Initial motivation: the app was not able to change behavior if the user did not already possess the initial motivation to quit. This was a key point mentioned across all data sources as described in the following statement:

*If I was on the path of trying to quit and desperately trying [to quit] then the game would help me, but due to not being in that mindset it did not [help me].*

Physical distraction: the apps could act only as a supplementary tool in smoking cessation, as it was not able to address the physical side of the smoking habit. As is explained when one user said:

*I chew some chewing gum…[it] just gives me something to do with my mouth.*

This phenomenon reflects Pavlov’s Theory of Classical Conditioning, whereby the instinctive, unconditioned stimulus (the urge to smoke) is paired with a new, conditioned stimulus (drawing for the app) [25]. However, it is important to note that merely forming an association between smoking and the app was in itself insufficient to change health behavior. In order to do so, it should be paired with intrinsic motivation as was emphasised by our expert interviews. Prof Nicholson stated:

If the app hasn’t built up intrinsic motivation and the user hasn’t found their own motivation to continue, then the health behavior will revert if there is no intrinsic motivation.

#### Modifiers Common to the mHealth Applications

We identified 5 modifiers of the mHealth interventions as shown in Textbox 1.

### Drivers and Modifiers of Gamified mHealth Interventions

#### Driver 1: Perceived Behavioral Control

Our data indicated that breaking down challenges of changing health behavior into smaller milestones, helped to increase the perceived behavioural control (PBC) of the individual by increasing their control beliefs, illustrated in the following:

If the end goal is just to quit smoking it makes it so hard, but if you have a game it enthuses the idea of something to work towards and it can steadily reward or punish you by setting short term goals…

It has been further suggested that ‘Flow’ might be involved in shifting the locus of control from external to internal regulation, explaining how gamification might impact control beliefs and subsequently, PBC [26,27]. Achievements and rewards stimulated self-efficacy by providing a feedback mechanism, and thus a form of performance monitoring [28]. In this way, the conditional rewards would reinforce positive health behavior and in turn serve as a conditioned stimulus [29,30]; illustrated with the following users’ statements:

The (achievements) felt good… achieving something… makes you feel like you can do it.

It constantly reminds you, it’s like going on a streak, you feel proud of yourself.

The game (achievements) showed that I can resist sometimes and proved that I can resist.

Participants exhibited anxiety at the prospect of going down a level if they were to smoke a concept known as loss aversion [31]:

It’s so annoying when you go down a level, I want to go up not down. I didn’t think much about gaining levels but I really did not want to lose levels.

It is essential to balance loss aversion against the possibility of negatively impacting self-efficacy by going down a level, and consequently reducing PBC.

PBC was also impacted by external influences, namely local networks (family, friends, and near acquaintances). If they did not support the idea of smoking cessation, it decreased self-efficacy, and thus PBC.

He [my husband] actually thinks that it’s possibly not the best time to do it [quit] because I have my exams coming in… so not go without any because… you’re going back to cigarettes.

This produced a negative effect as they discouraged the use of the intervention. Therefore, the local network had a profound effect in defining the level of perceived self-efficacy and their involvement should be minimized.

#### Driver 2: Intrinsic Motivation

Participants using the gamified intervention demonstrated greater levels of motivation and subsequent engagement than the nongamified cohort. Game elements such as rewards and level progression acted as motivational affordances leading to engagement. Participants in the nongamified cohort specifically mentioned game components with statements echoing the following quote: “If you put anything into a game it makes it more fun, and achieving something makes it more fun.”

Self-determination theory defines intrinsic motivation as ‘an activity one does because it is inherently enjoyable’ and extrinsic motivation as ‘doing something because it leads to a separable outcome’ [32]. The motivational effectiveness of extrinsic rewards will reduce over time; in the context of smoking this increases the likelihood of relapse as engagement decreases [33]. However, intrinsic motivation leads to increased frequency of behavior, and therefore increased engagement with the app [34].

Intrinsic motivation was also impacted by external influences. If local networks supported the idea of smoking cessation, participants were more likely to receive words of encouragement regarding their progress. Studies have shown positive feedback to be associated with smoking cessation, with the reverse also being true [35]. However, although words of encouragement from peers can lead to positive behavior change in the short term, the effects are unlikely to be lasting if the encouragement is not self-relevant. Reliance on extrinsic rewards, which create a sense of duty, should be avoided; especially if they are perceived as controlling:

The pressure does not help, you don’t want to be told what to do, you want to do it on your own merit and I want to quit when I want to. It feels very parental and ...having people shove their own ideas down my throat as if I am not aware of what I am doing is very patronizing.

Any such rewards will undermine the game and impact negatively on intrinsic motivation, thereby compromising engagement [32,36].

#### Modifiers of Gamified mHealth Interventions

There are 7 modifiers that have determined the strength of the drivers specific to gamification: (1) personalization, (2) meaningful framing, (3) challenge-ability balance, (4) unpredictability, (5) user-centered design, (6) fun, and (7) social community.

##### Personalization

Participants cited that achievements of the gamified intervention lacked self-relevance *:* “Smoking is personal and should not have premade incentives, people should generate their own incentives and the app should empower [them].” The orientation of the individual affects how they will perceive extrinsic rewards; whether they perceive it as controlling (externally oriented), or informational (internally oriented) [32]. An element of personalization can tailor an intervention to the individual, and thus account for an externally oriented user.

##### Meaningful Framing

Framing a challenge in a meaningful way works synergistically with the gamified reward system to enhance intrinsic motivation [37].

##### Challenge-Ability Balance

Ensuring a dynamic balance between the participants’ perceived ability and the perceived challenge is a core tenet of flow [36].

##### Unpredictability

Participants exhibited tedium after using the gamified intervention for some time, which led to disengagement *:* “Achievements became slightly repetitive and need to be more creative.” However, integrating variable rewards, which are informational and unpredictable in nature has been found to increase focus and engagement [38]. In contrast, rewards that are contingent on engagement and performance alone should be minimized as far as possible, where do they not align with the individual’s intrinsic motivation, or they risk undermining it [27].

##### User-Centered Design

Ensuring that the user’s needs and goals are the primary consideration at every stage of the process [39].

##### Fun

A common request from participants in the nongamified cohort was to add an element of fun to the game: “If it was a game with milestones and achievements and levels it would definitely be really cool… if it was a game I would definitely do that.” Fun can be defined as a type of intrinsic motivation that may play an important role in achieving a state of flow [40]. It is important to note that ‘fun’ is the product of the relationship between an activity and an individual’s goals, rather than solely as a property of the activity itself.

##### Social Community

Users were unwilling to share their progress via Facebook and Twitter. With multiple participants sharing the sentiment in the following quote: “I actually think it [sharing on social media] is counterproductive. You do it for yourself, not other people.”

However, they expressed desire to interact with like-minded individuals, with whom they could better relate. Kwon et al [41] reinforced these observations when they found that the motivations for social networking, and the motivations for building up reputation were not mutually exclusive.

## Discussion

### Framework

**Figure 4:**
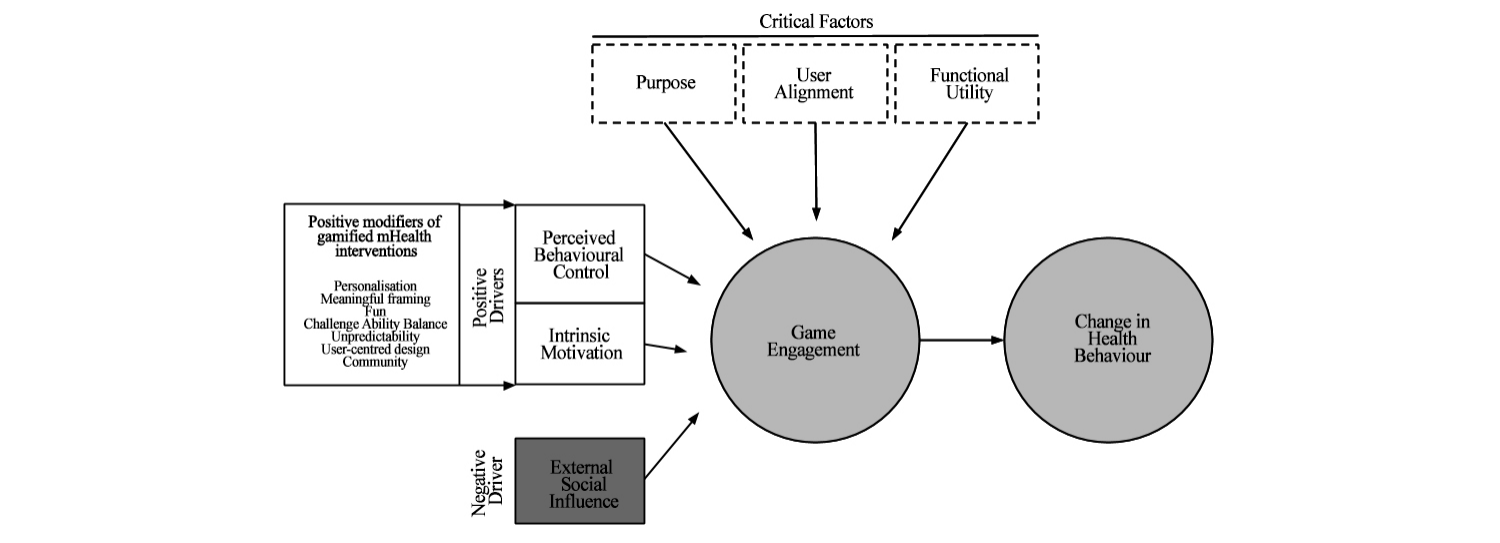
How positive health behavior can be promoted by the gamification of mHealth apps.

In this study, we compared 2 apps, 1 gamified and the other nongamified, in a longitudinal qualitative study and then analyzed our findings in the context of expert opinions and the extant literature. We sought to establish how best to exploit gamification as an effective tool to build and maintain engagement of mHealth apps designed to promote smoking cessation. This work culminated in the development of a framework to isolate the drivers and factors that govern effective gamification (Figure 4). The framework suggests that a change in health behavior is dependent on the degree of engagement with the gamified intervention and that this was influenced by ‘critical factors’ and ‘drivers’ of game engagement.

### Critical Factors

Critical factors were the 3 components that had to be present in order for users to engage with the game; absence of any one of these critical factors would lead to disengagement. A mHealth app looking to promote positive health behavior change needs a ‘purpose’ that is made explicit and clear to the user. However, this ‘purpose’ needs to align with the user’s own personal objective (‘user alignment’). This ‘user alignment’ is key to tapping into the user’s intrinsic motivation, ensuring sustained engagement with the intervention as explained by both experts and users alike.

The final critical factor is ‘functional utility’ or the perceived ability of the intervention to fulfil the needs and solve the problems of the participant. This was the most frequently coded code during the thematic analysis, with 39% (39/100) of codes referring to at least 1 of game or app functional utility. We found that when users’ perceived functional utility of the intervention was low, they became disengaged. To enhance functional utility, the intervention needs to be easy to use, designed around the user, and integrate a feedback mechanism to allow users to track their progress.

#### Drivers

We identified ‘perceived behavioral control’ and ‘intrinsic motivation’ as positive drivers, which when present, directly led to game engagement. In the context of health behavior, game engagement can be maximized by taking advantage of modifiers that boost self-efficacy and minimize control beliefs. We also observed intrinsic motivation to be a principal driver of game engagement, and should be maximized, by using the modifiers in the presence of the 3 critical factors.

The impact of positive drivers is determined by factors in the framework classed as modifiers. We identified 7 modifiers of gamified mHealth interventions: (1) personalisation: challenges and rewards that are self-relevant, (2) meaningful framing: link the challenge of changing health behavior to an overall self-relevant goal, (3) challenge-ability balance: a dynamic balance must exist between the perceived ability and perceived challenge, (4) unpredictability: unexpected rewards are perceived as least controlling types of rewards, (5) user-centered design: ensuring the user’s needs and goals are constantly met, (6) fun: the experience must be innately enjoyable, and (7) social community: create a community of like-minded individuals, where posting accolades will boost an individual’s reputation.

We also identified ‘external social influence’ as a negative driver, which should therefore be minimized to optimally promote positive health behavior. We observed that the presence of an external social influence negatively impacted self-efficacy and consequently decreased the individual’s perceived behavioral control and intrinsic motivation. For emphasis, ‘external social influence’ has been depicted to directly impact game engagement, although it does this by impairing the user’s PBC and intrinsic motivation.

#### Applicability of the Framework

Our aim is for our framework to be used as a guide for health care professionals and app developers in appraising whether a gamified app has the right ingredients to be successful in generating and promoting positive health behavior change. The successful implementation of gamified mHealth interventions will require a multidisciplinary approach, marrying input from clinicians, behavioral scientists, and game designers to build compelling apps [42]. As such, a further application of this framework is to provide a theoretical basis around which the multidisciplinary teams could collaborate.

### Limitations

Limitations to our study mainly relate to the infancy of gamification as a field, meaning only a limited number of interventions were available to us. Although the gamified intervention was identified as one of the leading apps implementing game mechanics, it fell short in a number of areas leading to a drop in engagement over time. However, it is unclear whether this was due to shortcomings solely within the app, or gamification itself. In order to fully understand the effect of a gamified intervention, we would have benefited from a more optimal implementation of gamification as well as testing interventions employing a wider range of game elements reflecting the variety of gamification strategies employed by different health apps. Moreover, while we tried to choose 2 apps that employed the same intervention content, bar the presence of gamified features in one and the absence in the other, there may well be some confounding variables responsible for our results that we were unable to identify in our analysis. To combat this, a further study would be required involving the creation, from scratch, of 2 interventions offering the same educational content and differing only by the use of gamified features. In addition, we were unable to examine the long-term impact of gamification beyond the 5-week study period. A more representative analysis of the overall smoking demographic could have been conducted with the inclusion of the diseased population and a larger sample of smokers from different geographical and socioeconomic contexts. A larger sample may also help to better elucidate the scale of the findings we present in the framework, for instance the extent to which external social influences result in a truly negative effect and whether there are instances where they may bolster an individual’s intrinsic motivation for example.

### Policy Implications of Implanting Gamification in the National Health Service

Offering individuals a gamified mHealth intervention for smoking cessation could be the answer to the inability of current NHS smoking cessation services to serve the population, particularly for millennials who have grown up as ‘digital natives’ [43]. A gamified mHealth intervention would confer the benefits of evidence-based behavioral therapy, while transforming the expensive interface of patient-doctor consultations, to one between patients and an app. Furthermore, the intervention will always be close at hand to the user helping to provide support when high-risk situations arise.

Gamified mHealth interventions should not be used in isolation, but rather be considered as an additional tool in the delivery of health care. For example, implementation among older, less technologically competent patients will prove challenging, with certain patients still favoring human-human interaction. As such, it will be important to continue to offer conventional behavioral support alongside a new intervention to optimize the effectiveness of the service.

### Conclusions

Gamification holds the potential for low-cost, highly effective mHealth solutions that may replace or supplement the behavioral support component found in current smoking cessation programs. Our proposed framework has been built on evidence specific to smoking cessation. We propose that it can also be extended to pave the way toward new methods of public health education, as our findings showed that gamification could be an effective modality for engaging people with the provision of information. However, questions still remain in relation to the long-term effects of gamification. Future research is required to evaluate the effectiveness of the above framework against current behavioral interventions in smoking cessation.

## Appendix

Multimedia Appendix 1 Semi-structured interview questions.

Multimedia Appendix 2 Thematic coding summary with codes and illustrative quotes regarding the expert interviews.

Multimedia Appendix 3 Thematic coding summary with codes and illustrative quotes regarding the Drivers for mHealth interventions. Quotes taken from both participant and expert interviews.

Multimedia Appendix 4 Thematic coding summary with codes and illustrative quotes regarding drivers for gamified mHealth interventions.

Multimedia Appendix 5 Participant concerns with illustrative quotes regarding gamified mHealth interventions.

Multimedia Appendix 6 British Thoracic Society Conference presentation December 2015.

## Abbreviations

NHS: National Health Service
PBC: perceived behavioral control
