# Supplementary material for: Game On? Smoking Cessation Through the Gamification of mHealth: A Longitudinal Qualitative Study
Source: JMIR Serious Games. 2016 Oct 24;4(2):e18. doi: 10.2196/games.5678 (PMC5099502; doi:10.2196/games.5678)
Supplement: Multimedia Appendix 1 [file games_v4i2e18_app1.pdf]

APPENDIX 1

Semi-structured interview questions.

Week 0: These are only to be used as a guideline. Ensure topics are fully explored.

- *Demographics:*
  - What is your name?
  - How old are you?
  - What is your ethnicity?
  - Record Gender
  - What is your job?
- *Smoking background:*
  - How many cigarettes do you currently have a day?
  - What brand of cigarettes do you smoke? What brand? What is the cost of a pack?
  - How long have you smoked for? How many a day?
  - What does smoking mean to you?
  - Do you call yourself a smoker?
  - What things do you enjoy about smoking?
  - And what things don’t you like about smoking?
  - Are you trying to quit smoking?
  - What techniques are you currently using? How long they are using them for?
  - Have you ever tried to stop smoking? What drives your see to want to quit?
    - IF YES:
    - About how many times have you tried to stop smoking?
    - What made you think about stopping smoking?
    - What happened when you tried to stop?
    - How did you try to stop? PROBE FULLY patches/ ecigarette/ etc

- Why did it not work?
- IF NO:
- Has anyone ever encouraged you to stop smoking?
  - IF YES:
  - Who has encouraged you to stop smoking?
  - How did you react to these suggestions?
  - Why you react the way you did?
  - What does quitting smoking mean to you?
  - What would you gain about not smoking?
  - What would you lose?
  - What would you miss the most when quitting?
  - What items of technology do you use? How often?
  - Do you ever play games on the technology devices you use? IF YES how often?
  - Have you ever played games on a technological device? IF YES how often did you play?

Week 1,2,5: These are only to be used as a guideline. Ensure topics are fully explored.

- *Progress:*
  - How many cigarettes have you smoked per day this week?
  - How did you feel when you smoked?
  - How does this feeling differ from before?
  - Is there any particular reason that you have been smoking this week?
  - How have your stress levels been this week?
  - How often did you use this app?
  - How stressed have you been over the last week?
- *Experience using the App:*
  - Can you please tell me about your experience using the app?
  - Did you find the app useful?

    - IF YES: How did you feel this app helped you?
        - What did you like about the app?
        - What did you not like
- *App effect on behaviour:*
    - Did you notice any change to your behaviours as a result of the app?
    - Have you started using any new techniques to quit smoking in the last 2 weeks? (patches, eCigs)
        - IF YES: Why?
    - What impact did the information the app gave to you have on you?
    - What information did you find most useful?
        - How would you rank them in order of importance to you? Why?
- *App effect on feelings:*
    - Has the app had any effect on your willpower to stop?
    - How do you feel about sharing progress?
    - Did you share your progress via social media?
        - IF YES: How did you feel when you shared your progress? Why did you share your progress?
        - IF NO: Why did you not share your progress?
    - Did you share your progress in any other way? i.e. discussing with friends etc.
- *Kwit 2 Specific:*
    - What did you think about the profile page? Was it motivating?
    - How did you feel when you went down a level?
    - Did the prospect of going down a level influence your decision to smoke?
    - How did you feel when you reached a new level?
    - How did you feel when you reached a new achievement?
    - The achievements get harder, how does that make you feel?
    - Have you compared your achievements with other users using the app? How did you feel? Did that motivate you?

- Did the game affect your motivation?

Exit Interview: These are only to be used as a guideline. Ensure topics are fully explored.

- How motivated were you at the start of the study to quit smoking?
- How motivated are you now to quit smoking?
- What did you think of the app?
- Did you like the app?
- What did you not like about the app?
- What has been the perception of others to you quitting smoking?
- *Gamification Specific:*
    - What did you think of the game concept?
    - How easy is app to use? Is it easy to navigate?
    - Did the app get repetitive?
    - If YES how would you make it less repetitive?
    - Do you think mobile apps are an effective way to help stop smoking?
    - Do you think that the concept of using games to help people stop smoking is / could be effective? Why?
    - What do you think is the most effective way to help smokers quit?
    - Do you think that you are a person who is health conscious?
    - If no what type of health risky behaviours do you partake in?
    - Do you believe you have an addictive personality?
    - Are you a user of any illicit drugs or substances?
    - Do you gamble? If YES: How often?
- *Progress*
    - Experience using the app
    - Effect of app on behaviour
    - Effect of app on feelings
    - What they like about the app
    - What they don't like
