# Supplementary material for: Game On? Smoking Cessation Through the Gamification of mHealth: A Longitudinal Qualitative Study
Source: JMIR Serious Games. 2016 Oct 24;4(2):e18. doi: 10.2196/games.5678 (PMC5099502; doi:10.2196/games.5678)
Supplement: Multimedia Appendix 2 [file games_v4i2e18_app2.pdf]

**APPENDIX 2**

**Table 2 - Thematic coding summary with codes and illustrative quotes regarding the expert interview findings.**

| Code | Quote | Label |
|---|---|---|
| Gamification can only reinforce desired behaviours. | “Gamification can be very effective in influencing health behaviours IF an individual is already highly motivated to change their behaviour. I do not think that gamification can generate a motivation that does not already exist, [it] can only nudge [motivation].” | 2A |
| Gamified interventions need to address the user’s intrinsic motivation. | “‘It can work well in the short term, but is problematic for long-term change. Since rewards replace intrinsic motivation, people grow weary of the same rewards over time. If the app hasn’t built up intrinsic motivation and the user hasn’t found their own motivation to continue, then the health behaviour will revert if there is no intrinsic motivation” | 2B |
| Smartphone apps are an excellent platform for intervention. | “Also, since many people carry mobile devices with them throughout the day, mobile apps can be useful for more frequent interactions” | 2C |
| Critical success factors for game engagement include: personalisation, attractive interface and feedback. | “When we talk about gamification in terms of health outcomes, the real challenge has been trying to create things that look and feel as good. Are we going to be able to create a game that really engages people in the same way as Call of Duty or Minecraft? Evidence shows that all of these elements work.” | 2D |

| | | |
|---|---|---|
| | | |
| | “I have very inside knowledge, by knowing some of the guys at Apple working on healthkit. They’re creating it to collect data without you recognising it. That’s the holy grail of social science” | 2E |
| | “People aren’t very good at counting and measuring their performance so this looks pretty good” | 2F |
| Rewards need to be variable. | “Since rewards replace intrinsic motivation, and people grow weary of the same rewards over time, then people grow weary of a gamification system over time.” | 2G |
| Gamification can replace current behavioural therapies as an adjunct to pharmacotherapies | “So where I see this pitch now is an adjunct to the smoking cessation therapies out there. I think it’s more than that [replacement to behavioural support therapies], it's not just about replacing the professional but the point of care.” | 2H |
