# Supplementary material for: Game On? Smoking Cessation Through the Gamification of mHealth: A Longitudinal Qualitative Study
Source: JMIR Serious Games. 2016 Oct 24;4(2):e18. doi: 10.2196/games.5678 (PMC5099502; doi:10.2196/games.5678)
Supplement: Multimedia Appendix 3 [file games_v4i2e18_app3.pdf]

APPENDIX 3

Table 3: Thematic coding summary with illustrative quotes regarding Drivers for mHealth interventions. Quotes taken from both participant and expert interviews.

Driver	Code	Quote	Label
Attitude Change	Information useful for maintaining engagement	“I was quite aware of all the benefits but it is nice to have it all reiterated. Just to look at the information and see yeah this is the good I am getting from stopping smoking”	3A
	Notifications [Reminders] helped maintain engagement	“One a day during lunch time notifications are good, reminds me consciously trying to stop [smoking]., a visual reminder….now that I am actually getting these cravings the constant reminders are good”’	3B
	Tracking progress increased awareness of smoking [e.g. number of cigarettes smoked]	“The status bars, they give nice visuals. Like sometimes it’s hard to visualise exactly what a cigarette means but the bars help you visualise it”	3C
	Attractive interface increases salience of user’s behaviour	“Attractive platform may actually help to incentivise or encourage someone”	3D
	Personalising the implications of	“First of all contextualising the advantage of stopping smoking [personalisation]	3E

	smoking can aid behaviour change	gives them a scare, a bit of a shock… this is what happens in real life terms of making people want to quit smoking.”	
Goal Setting	Tracking progress increased motivation	“It made me much more conscious of how much I was smoking, it was good to track progress...It makes me think do I really need to smoke, is it worth it for what I’m going to lose, you don’t think about that normally but the app helps you”	3F
	Felt a sense of commitment to the app	“It feels like I’ve committed to this, so I am more motivated to try and make it work”	3G
	App made them feel guilty	“When I smoked it’s like cheating yourself, you betray yourself when you press the button...Now when I think about it I feel horrendously guilty [when I smoked].””	3H
Association	Psychological link between app and smoking	"The app helped me create an association between app and smoking"	3I
		“My routine has changed now, when I get my cigarette out I automatically get my phone out as well now. The app has become integrated into my smoking”	3J
