# Supplementary material for: Game On? Smoking Cessation Through the Gamification of mHealth: A Longitudinal Qualitative Study
Source: JMIR Serious Games. 2016 Oct 24;4(2):e18. doi: 10.2196/games.5678 (PMC5099502; doi:10.2196/games.5678)
Supplement: Multimedia Appendix 4 [file games_v4i2e18_app4.pdf]

APPENDIX 4

Table 4: Thematic coding summary with illustrative quotes regarding Drivers for Gamified mHealth interventions.

Driver	Code	Quote	Label
Perceived Behavioural Control	The game made them feel like they could quit	“If the end goal is just to quit smoking it makes it so hard, but if you have a game it enthuses the idea of something to work towards and it can steadily reward or punish you by setting short term goals so it would be more consistent and prevent relapses"	4A
		“The game showed me that I can resist sometimes, and proved that I can resist”	4B
		“But after feeling proud, I have had multiple instances where I have resisted the urge and that self- satisfaction is a strong feeling...Pressing the button focuses all those feelings on a particular moment.”	4C
	Achievements and/or levels motivated them to carry on	“It constantly reminds you, it’s like going on a streak. You feel proud of yourself”	4D
		“The positive feedback you get from resisting is much more than the negative feedback you get from pressing smoke.	4E

		Pressing smoke makes people feel a bit crap when managing their habit. Any other motivational similar to the resist part would help. Positive reinforcement is a lot better to help me push myself”	
	Prospect of going down a level made me stay away from smoking	"It's so annoying when you go down a level, I want to go up not down. I didn’t think much about gaining levels but I really did not want to lose levels"	4F
Intrinsic Motivation	Game elements led to increased motivation	“If you put anything into a game it makes it more fun, and achieving something makes it more fun”	4G
		“I loved the achievements like the money. It showed me a number that I never thought about”	4H
