# Supplementary material for: Game On? Smoking Cessation Through the Gamification of mHealth: A Longitudinal Qualitative Study
Source: JMIR Serious Games. 2016 Oct 24;4(2):e18. doi: 10.2196/games.5678 (PMC5099502; doi:10.2196/games.5678)
Supplement: Multimedia Appendix 5 [file games_v4i2e18_app5.pdf]

APPENDIX 5

Table 5: Participant concerns with illustrative quotes regarding gamified mHealth interventions.

Concern	Quote	Label
Generalised achievements leading to lack of personalisation and self-relevance	"Smoking is personal and should not have premade incentives, people should generate their own incentives and the app should empower [them]" **22 Y/O Male**	5A
	"Everyone's incentives are different, the achievements don't relate to me." **19 Y/O Male**	5B
Rewards and achievements eventually became tedious	"Achievements became slightly repetitive and need to be more creative" **33 Y/O Female**	5C
Social media was not the medium to share your progress	"I actually think it [sharing on social media] is counterproductive. You do it for yourself not other people." **38 Y/O Female**	5D
