# Supplementary material for: Game On? Smoking Cessation Through the Gamification of mHealth: A Longitudinal Qualitative Study
Source: JMIR Serious Games. 2016 Oct 24;4(2):e18. doi: 10.2196/games.5678 (PMC5099502; doi:10.2196/games.5678)
Supplement: Multimedia Appendix 6 [file games_v4i2e18_app6.pptx]

## Slide 1
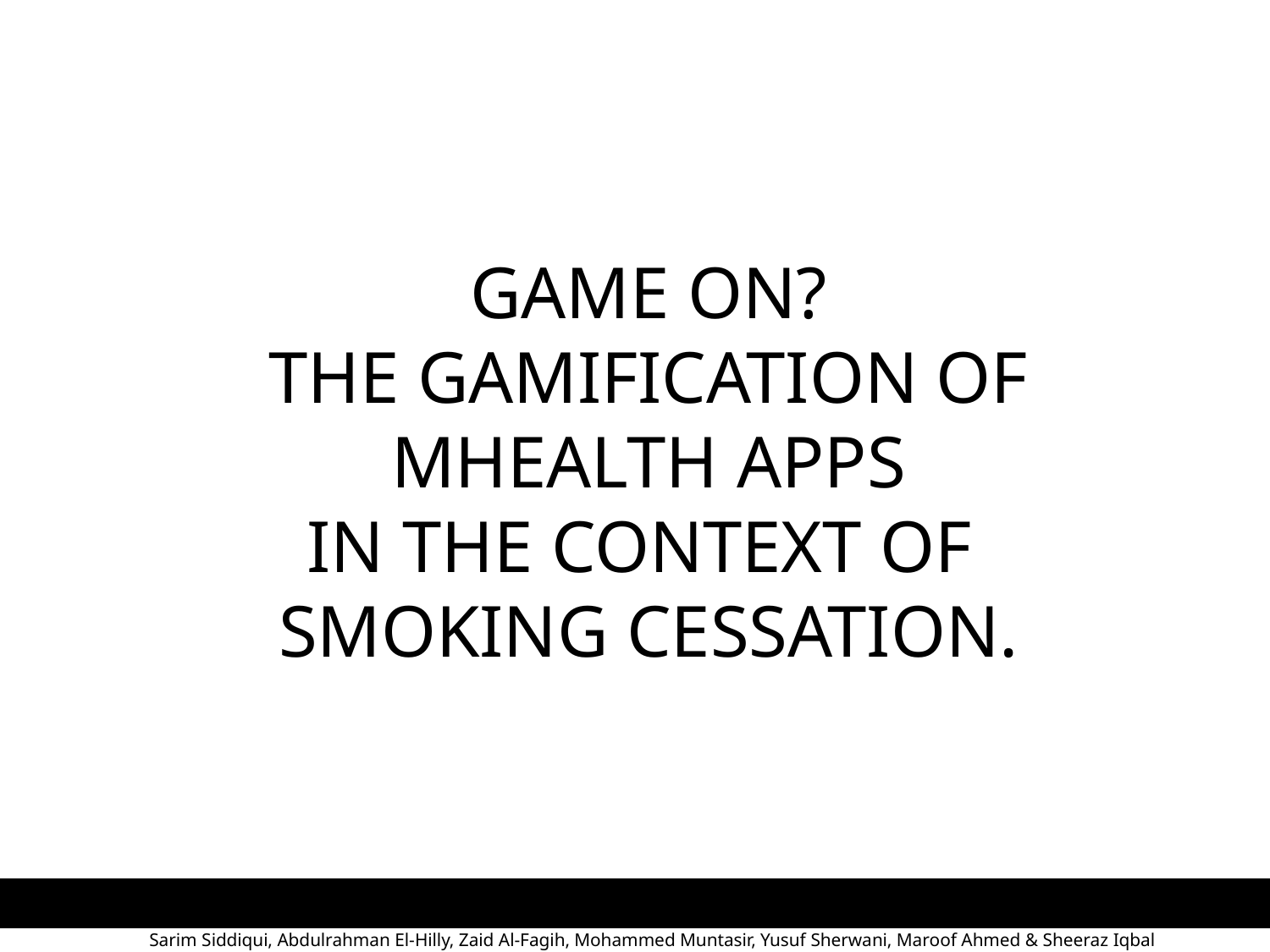

GAME ON?
 THE GAMIFICATION OF
MHEALTH APPS
IN THE CONTEXT OF
SMOKING CESSATION.
Sarim Siddiqui, Abdulrahman El-Hilly, Zaid Al-Fagih, Mohammed Muntasir, Yusuf Sherwani, Maroof Ahmed & Sheeraz Iqbal

## Slide 2
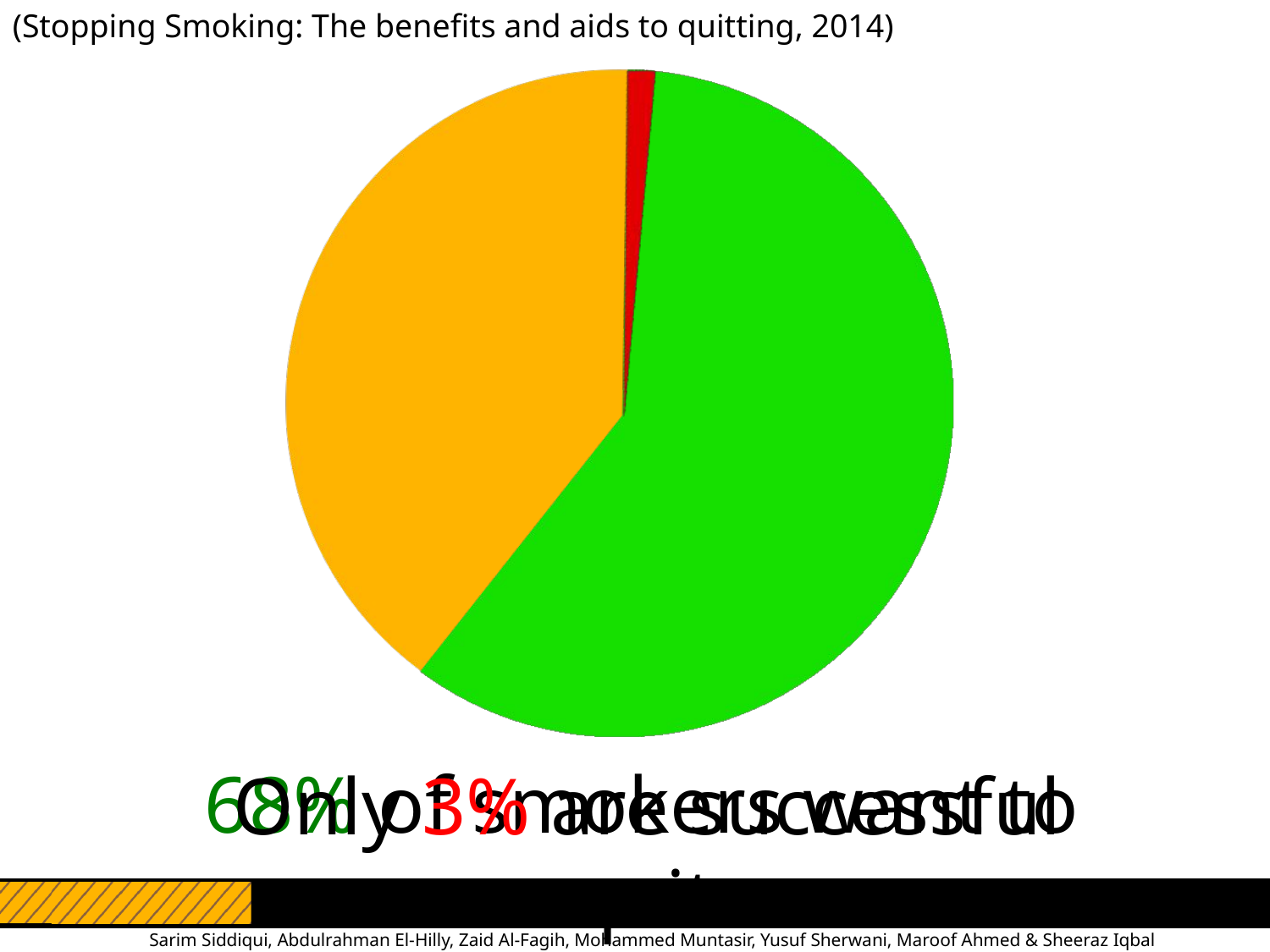

(Stopping Smoking: The benefits and aids to quitting, 2014)
68% of smokers want to quit
Only 3% are successful
Sarim Siddiqui, Abdulrahman El-Hilly, Zaid Al-Fagih, Mohammed Muntasir, Yusuf Sherwani, Maroof Ahmed & Sheeraz Iqbal

## Slide 3
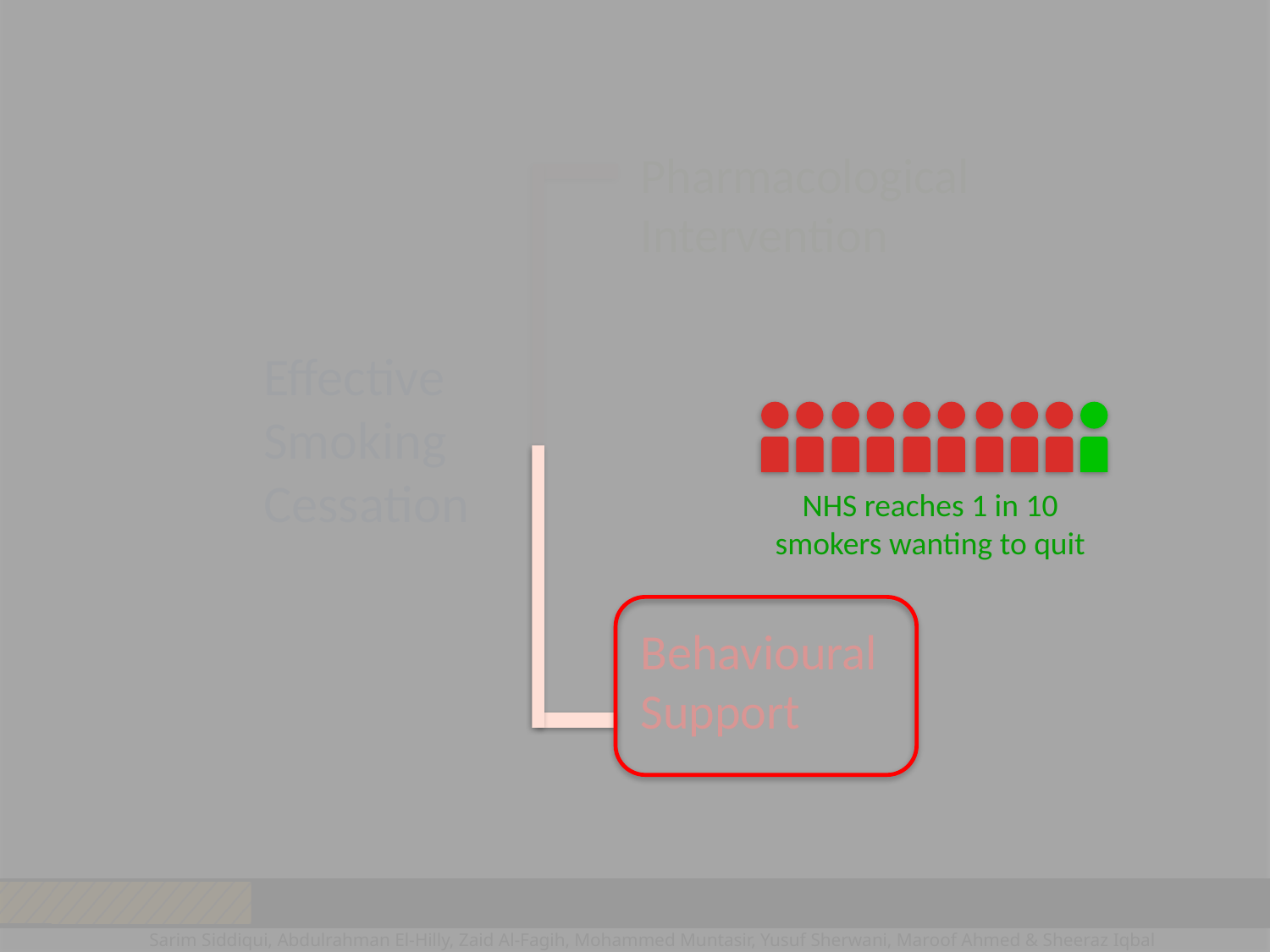

Pharmacological
Intervention
Effective
Smoking
Cessation
NHS reaches 1 in 10 smokers wanting to quit
Behavioural Support
Sarim Siddiqui, Abdulrahman El-Hilly, Zaid Al-Fagih, Mohammed Muntasir, Yusuf Sherwani, Maroof Ahmed & Sheeraz Iqbal

## Slide 4
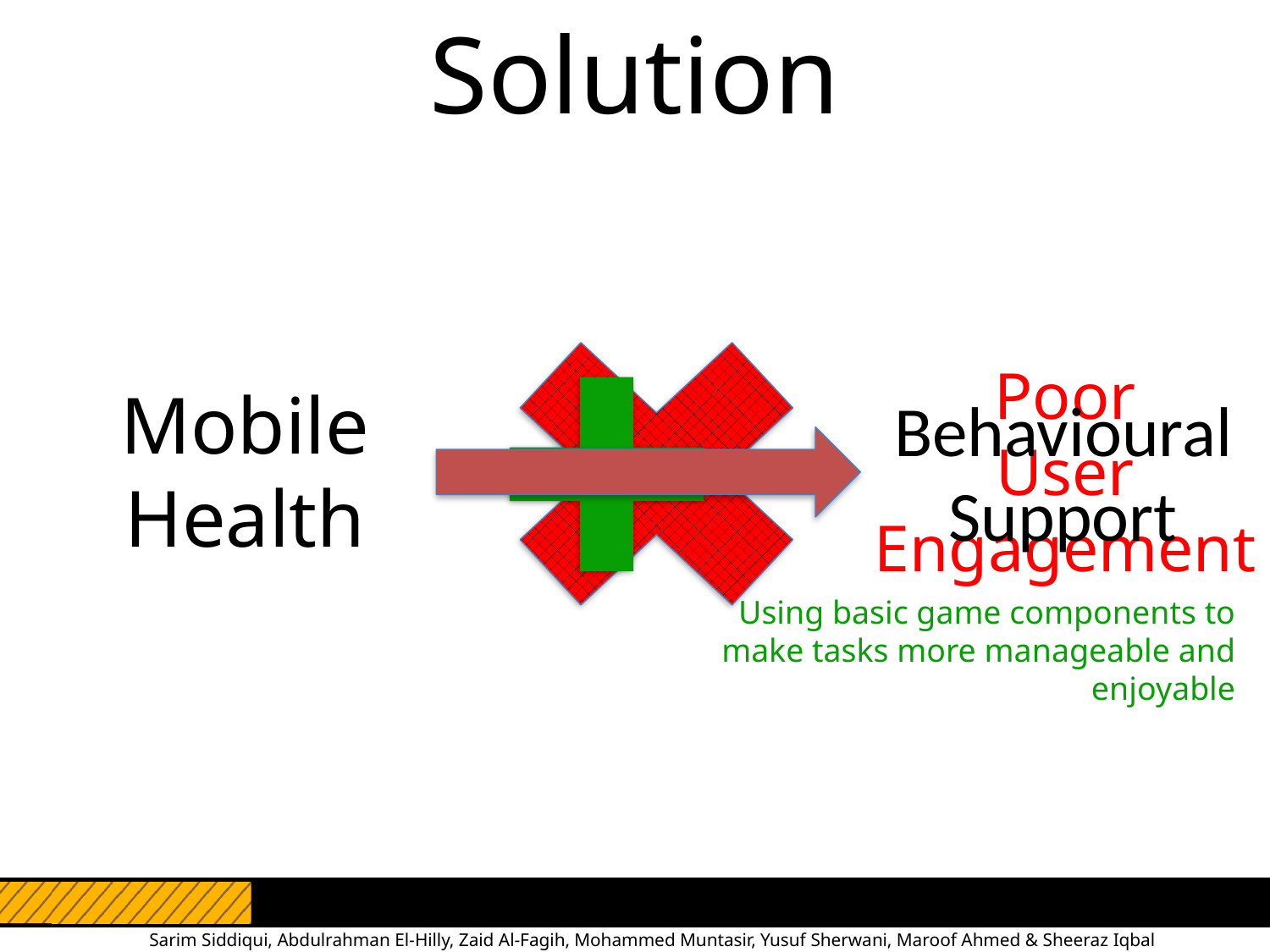

Solution
Poor
User
Engagement
Mobile
Health
Behavioural Support
Using basic game components to make tasks more manageable and enjoyable
Sarim Siddiqui, Abdulrahman El-Hilly, Zaid Al-Fagih, Mohammed Muntasir, Yusuf Sherwani, Maroof Ahmed & Sheeraz Iqbal
Gamification

## Slide 5
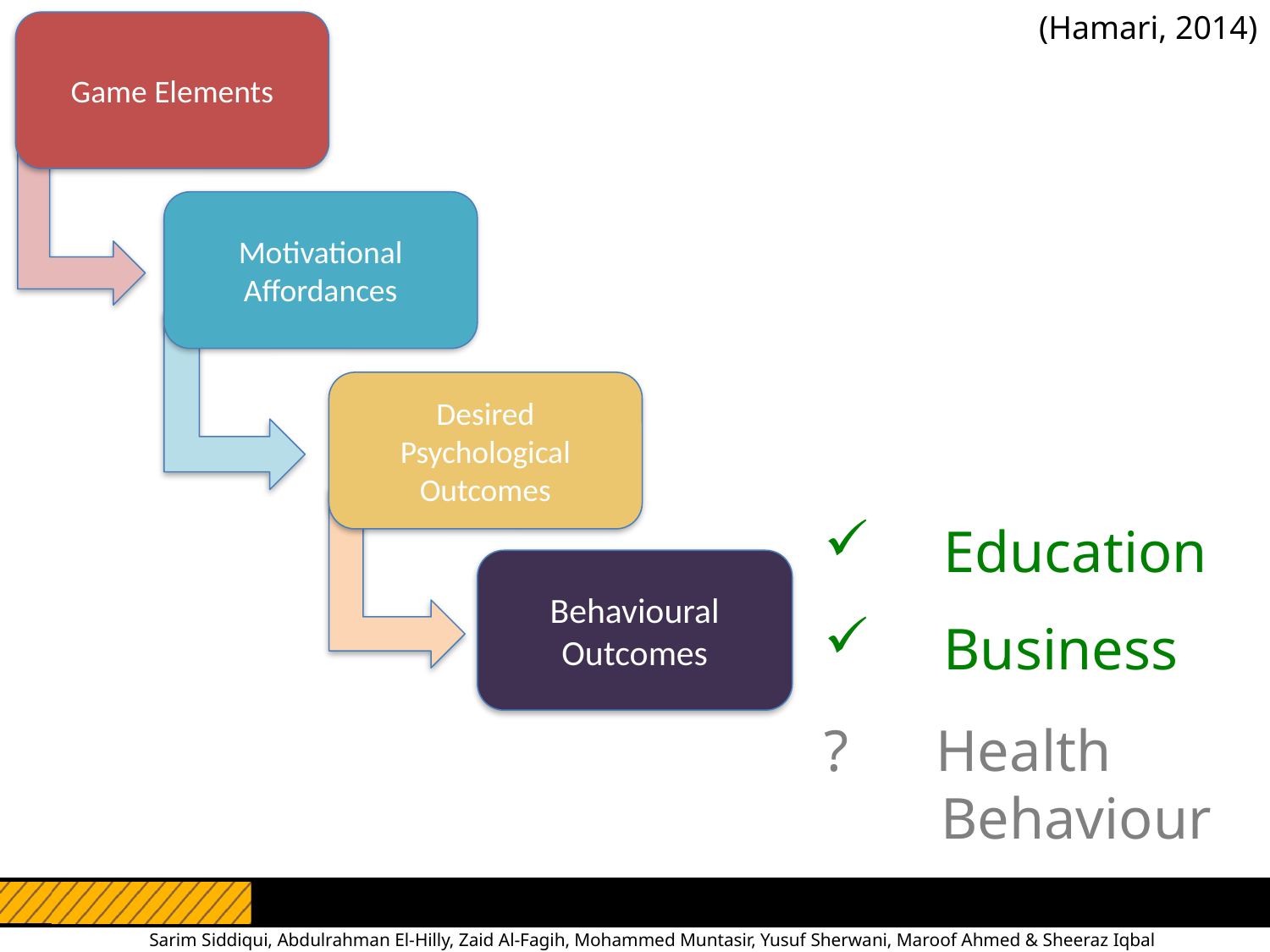

(Hamari, 2014)
Game Elements
Motivational Affordances
Desired Psychological Outcomes
 Education
Behavioural Outcomes
 Business
? Health
 Behaviour
Sarim Siddiqui, Abdulrahman El-Hilly, Zaid Al-Fagih, Mohammed Muntasir, Yusuf Sherwani, Maroof Ahmed & Sheeraz Iqbal

## Slide 6
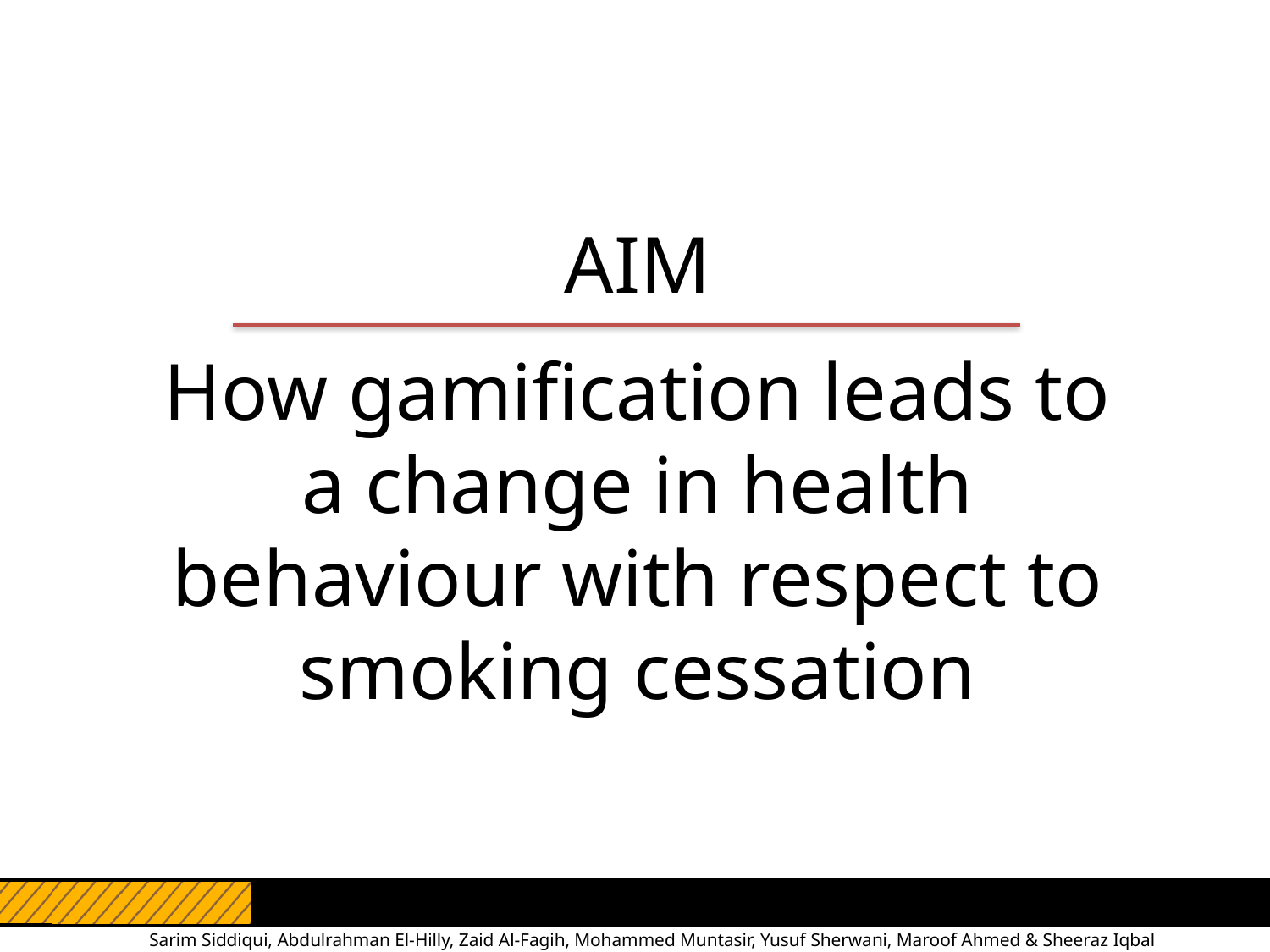

AIM
How gamification leads to a change in health behaviour with respect to smoking cessation
Sarim Siddiqui, Abdulrahman El-Hilly, Zaid Al-Fagih, Mohammed Muntasir, Yusuf Sherwani, Maroof Ahmed & Sheeraz Iqbal

## Slide 7
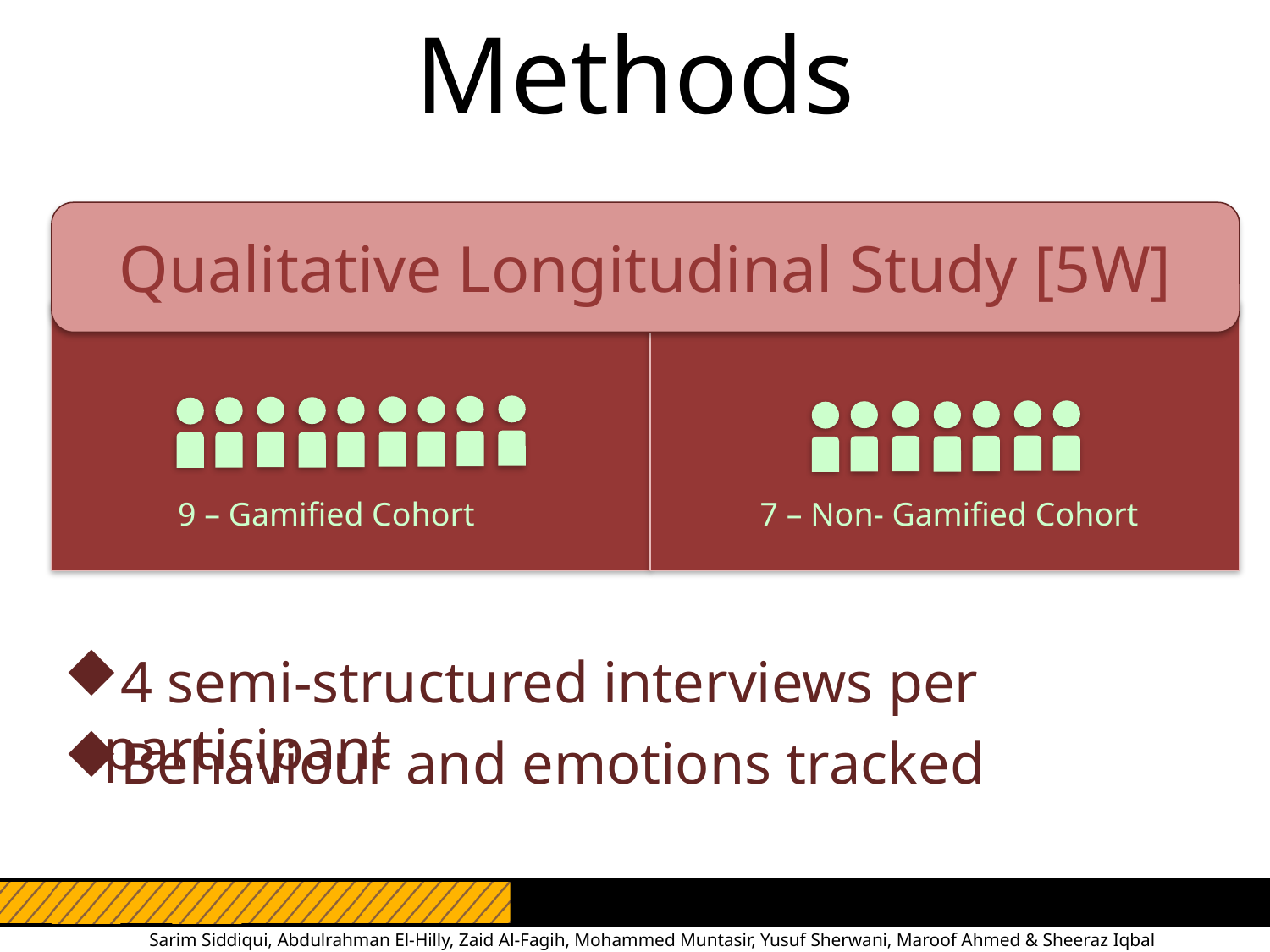

Methods
Qualitative Longitudinal Study [5W]
9 – Gamified Cohort
7 – Non- Gamified Cohort
4 semi-structured interviews per participant
Behaviour and emotions tracked
Sarim Siddiqui, Abdulrahman El-Hilly, Zaid Al-Fagih, Mohammed Muntasir, Yusuf Sherwani, Maroof Ahmed & Sheeraz Iqbal

## Slide 8
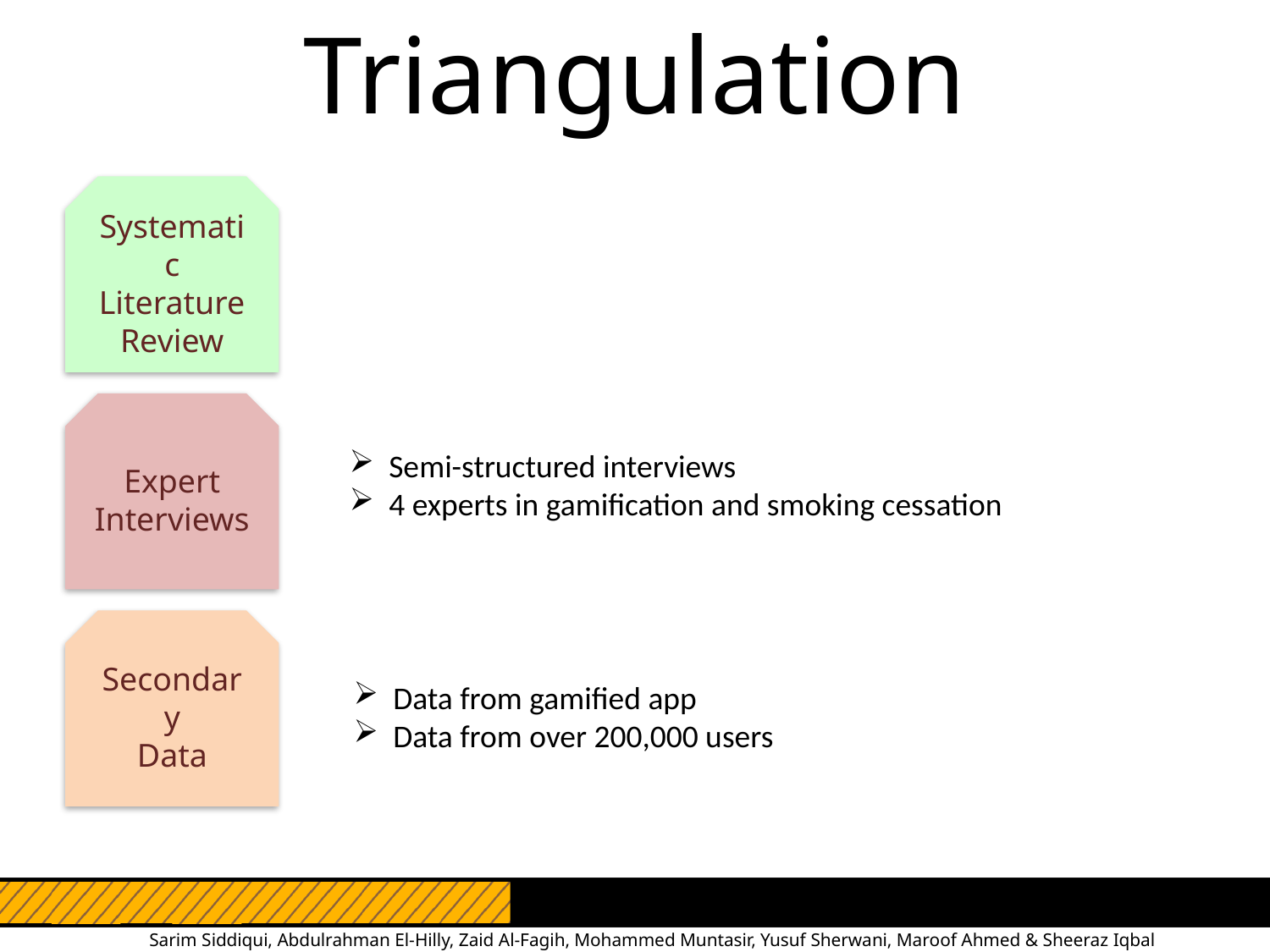

Triangulation
Systematic
Literature
Review
Expert
Interviews
Semi-structured interviews
4 experts in gamification and smoking cessation
Secondary
Data
Data from gamified app
Data from over 200,000 users
Sarim Siddiqui, Abdulrahman El-Hilly, Zaid Al-Fagih, Mohammed Muntasir, Yusuf Sherwani, Maroof Ahmed & Sheeraz Iqbal

## Slide 9
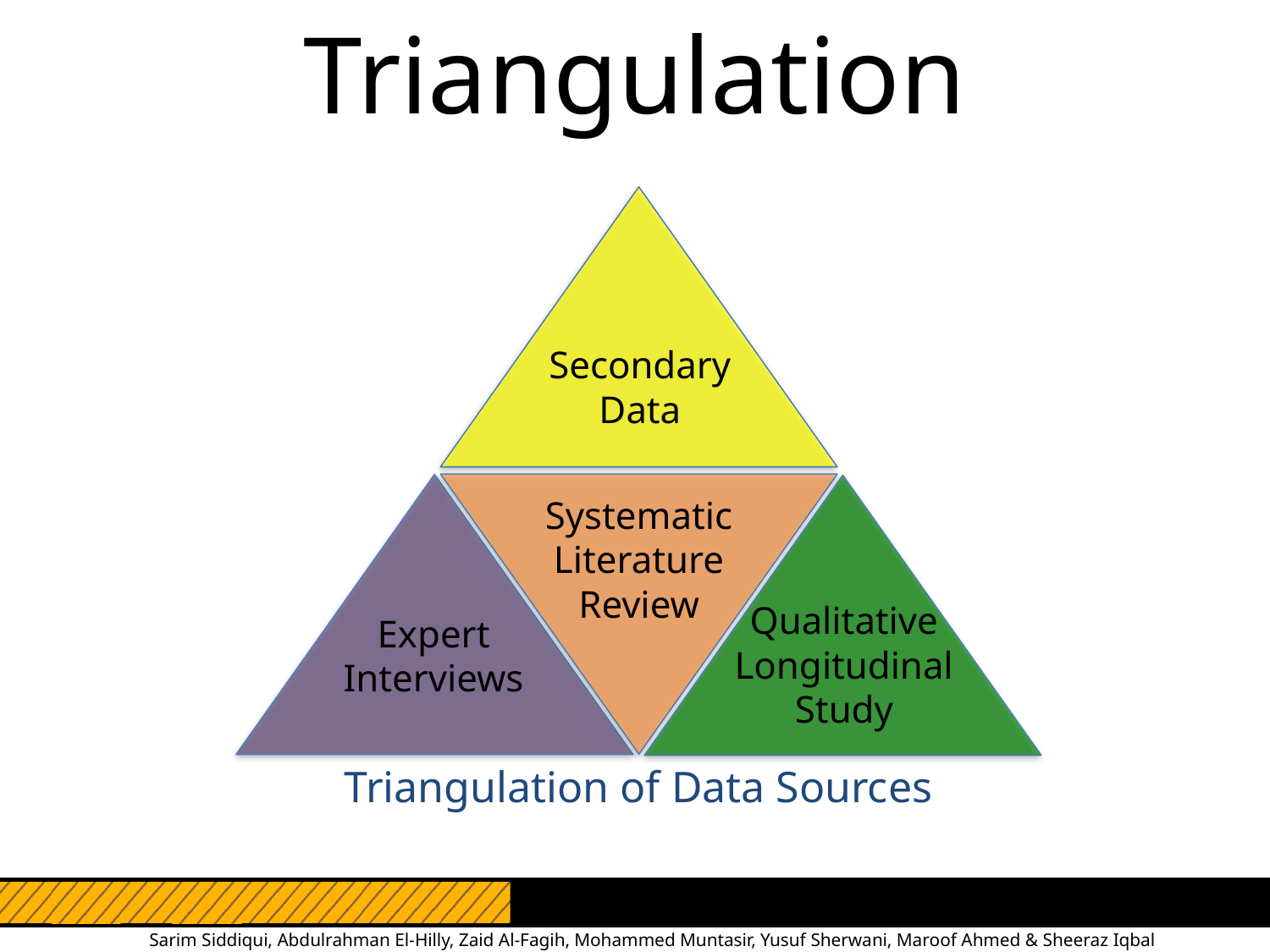

Triangulation
Secondary
Data
Systematic
Literature
Review
Qualitative
Longitudinal
Study
Expert
Interviews
Triangulation of Data Sources
Sarim Siddiqui, Abdulrahman El-Hilly, Zaid Al-Fagih, Mohammed Muntasir, Yusuf Sherwani, Maroof Ahmed & Sheeraz Iqbal

## Slide 10
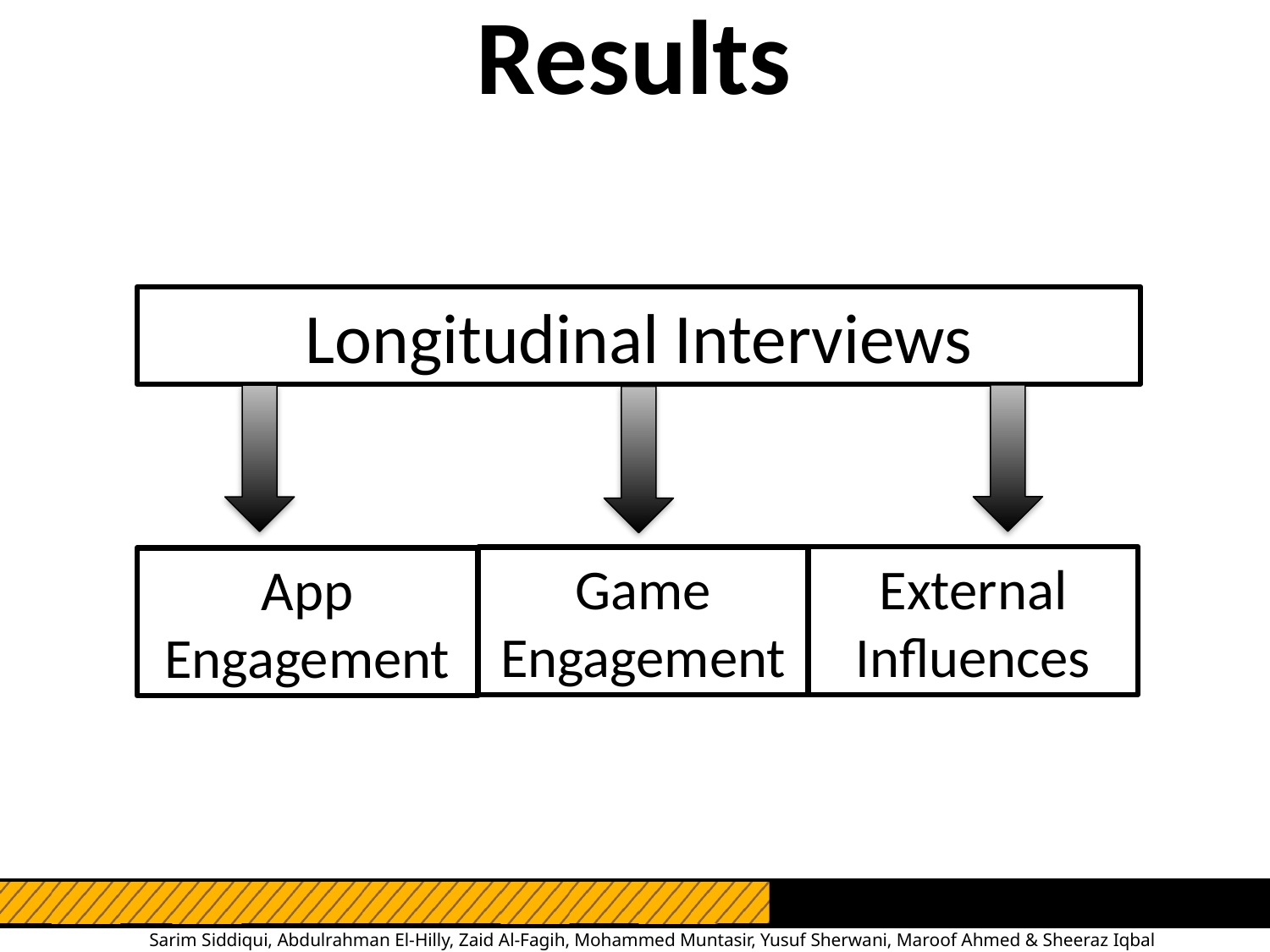

Results
Longitudinal Interviews
External
Influences
Game
Engagement
App
Engagement
Sarim Siddiqui, Abdulrahman El-Hilly, Zaid Al-Fagih, Mohammed Muntasir, Yusuf Sherwani, Maroof Ahmed & Sheeraz Iqbal

## Slide 11
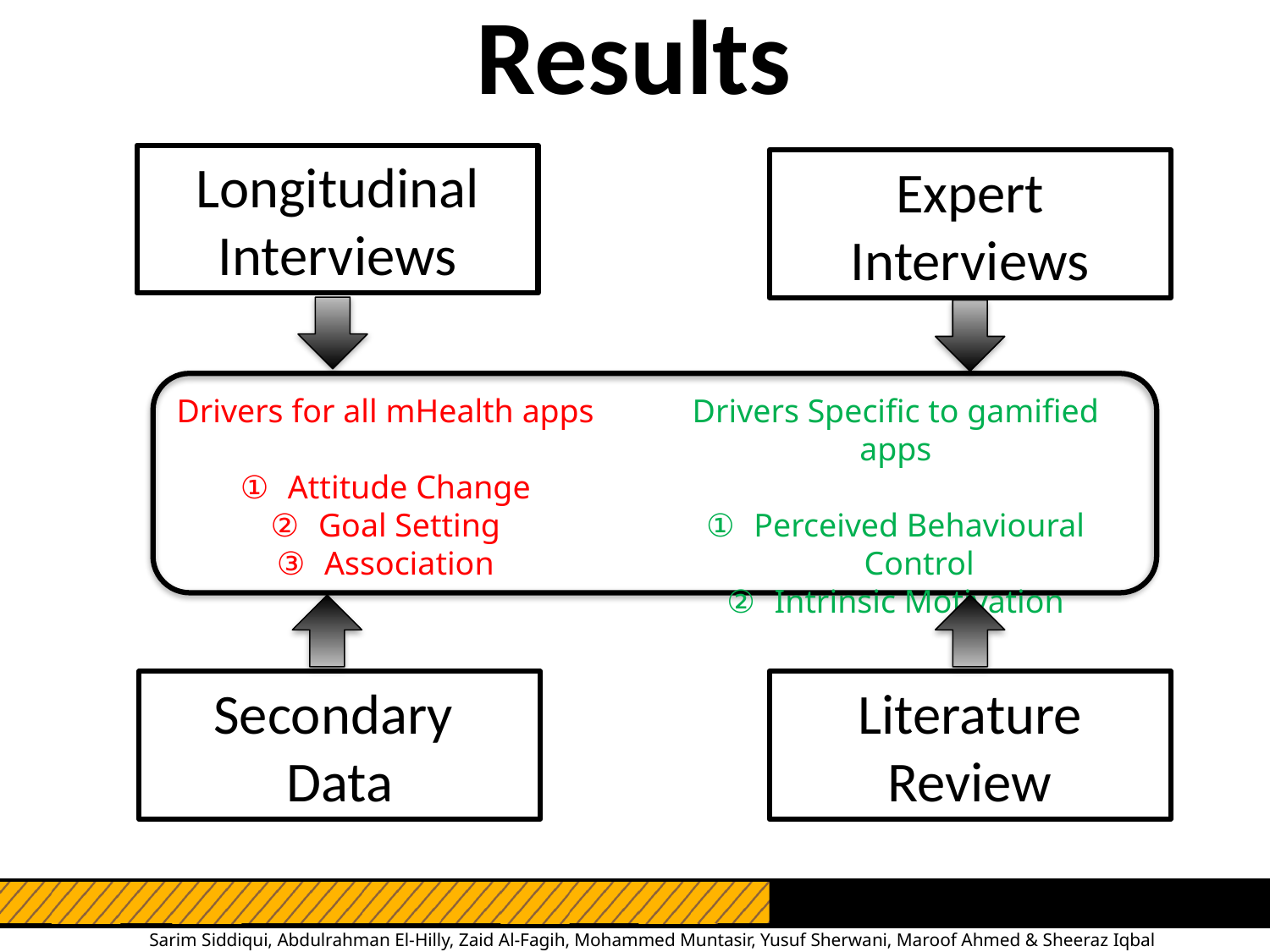

Results
Longitudinal Interviews
Expert Interviews
Drivers for all mHealth apps
Attitude Change
Goal Setting
Association
Drivers Specific to gamified apps
Perceived Behavioural Control
Intrinsic Motivation
Secondary
Data
Literature
Review
Sarim Siddiqui, Abdulrahman El-Hilly, Zaid Al-Fagih, Mohammed Muntasir, Yusuf Sherwani, Maroof Ahmed & Sheeraz Iqbal

## Slide 12
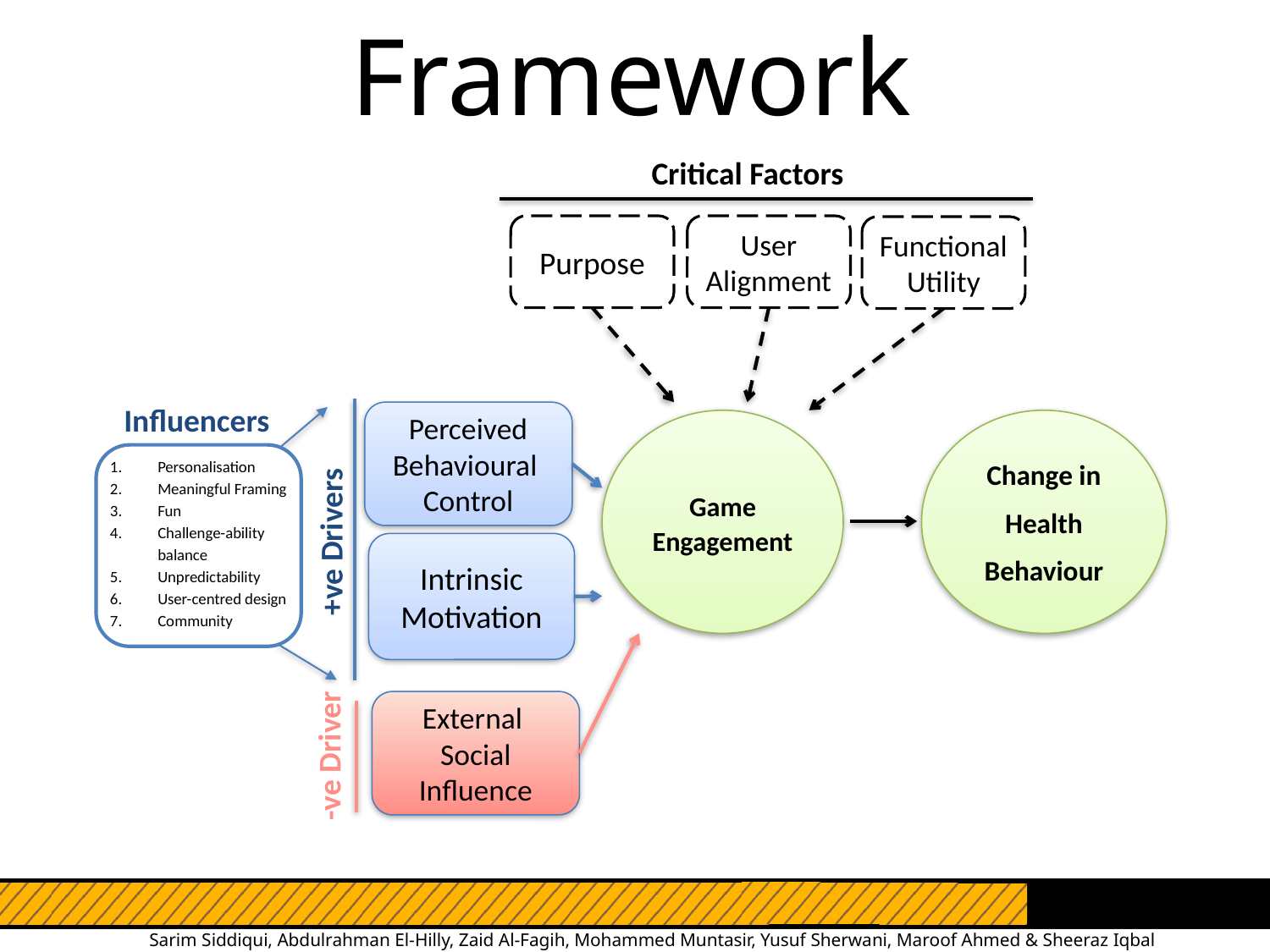

Framework
Critical Factors
Purpose
User Alignment
Functional
Utility
Influencers
Perceived
Behavioural
Control
Change in
Health
Behaviour
Game Engagement
Personalisation
Meaningful Framing
Fun
Challenge-ability balance
Unpredictability
User-centred design
Community
+ve Drivers
Intrinsic
Motivation
External
Social
Influence
-ve Driver
Sarim Siddiqui, Abdulrahman El-Hilly, Zaid Al-Fagih, Mohammed Muntasir, Yusuf Sherwani, Maroof Ahmed & Sheeraz Iqbal

## Slide 13
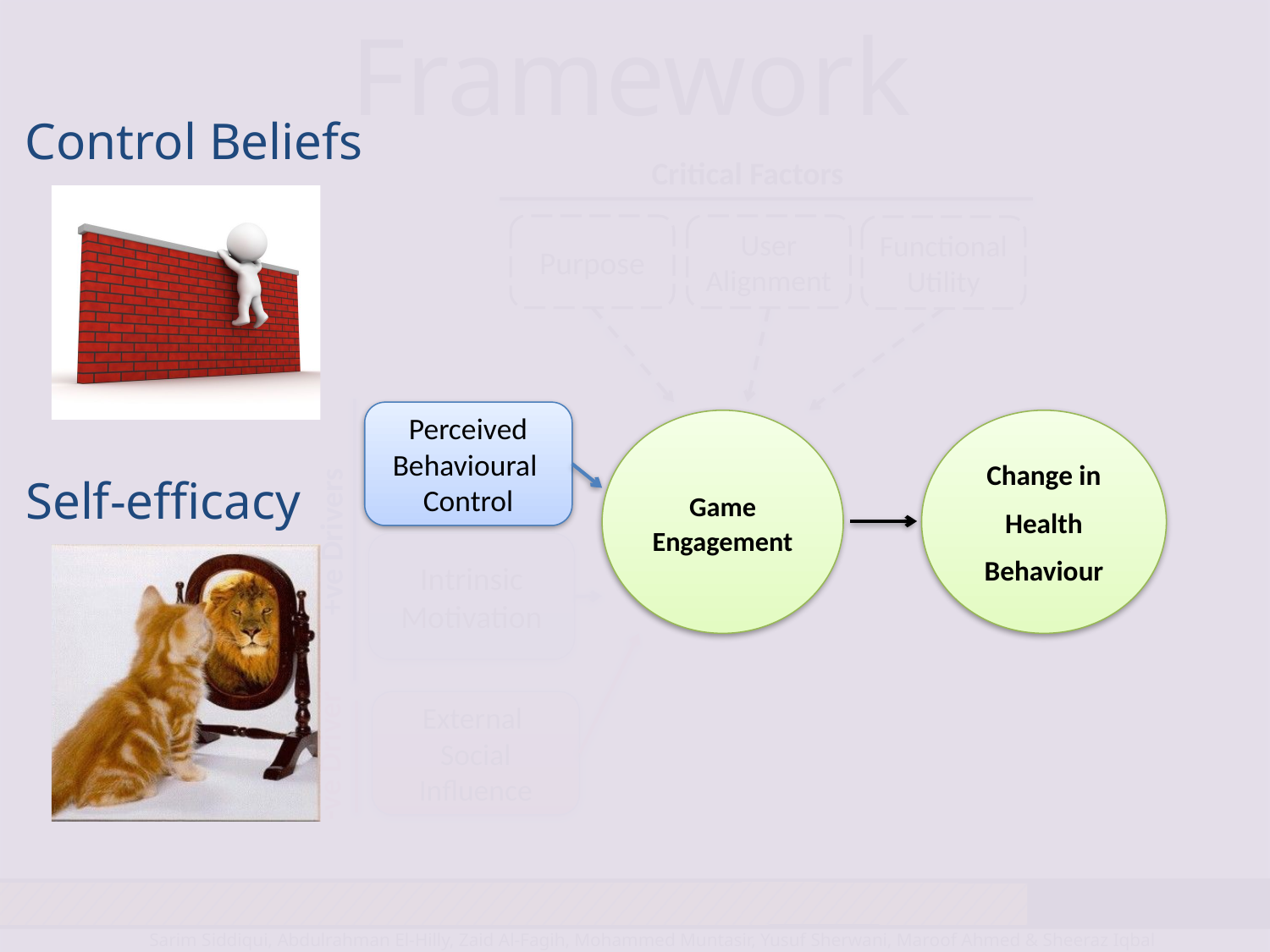

Framework
Control Beliefs
Critical Factors
Purpose
User Alignment
Functional
Utility
Perceived
Behavioural
Control
Change in
Health
Behaviour
Game Engagement
Self-efficacy
+ve Drivers
Intrinsic
Motivation
External
Social
Influence
-ve Driver
Sarim Siddiqui, Abdulrahman El-Hilly, Zaid Al-Fagih, Mohammed Muntasir, Yusuf Sherwani, Maroof Ahmed & Sheeraz Iqbal

## Slide 14
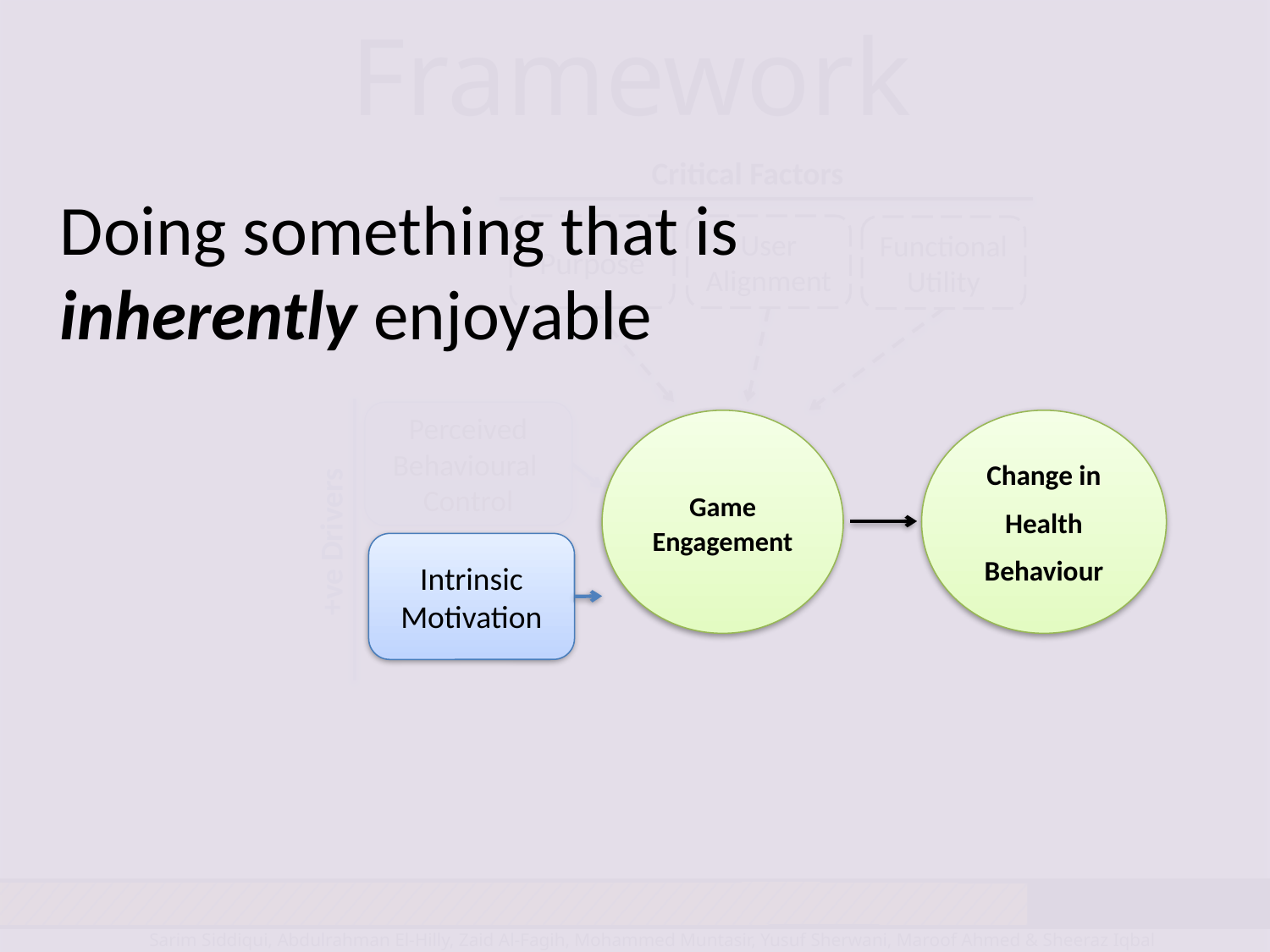

Framework
Critical Factors
Doing something that is inherently enjoyable
Purpose
User Alignment
Functional
Utility
Perceived
Behavioural
Control
Change in
Health
Behaviour
Game Engagement
+ve Drivers
Intrinsic
Motivation
Sarim Siddiqui, Abdulrahman El-Hilly, Zaid Al-Fagih, Mohammed Muntasir, Yusuf Sherwani, Maroof Ahmed & Sheeraz Iqbal

## Slide 15
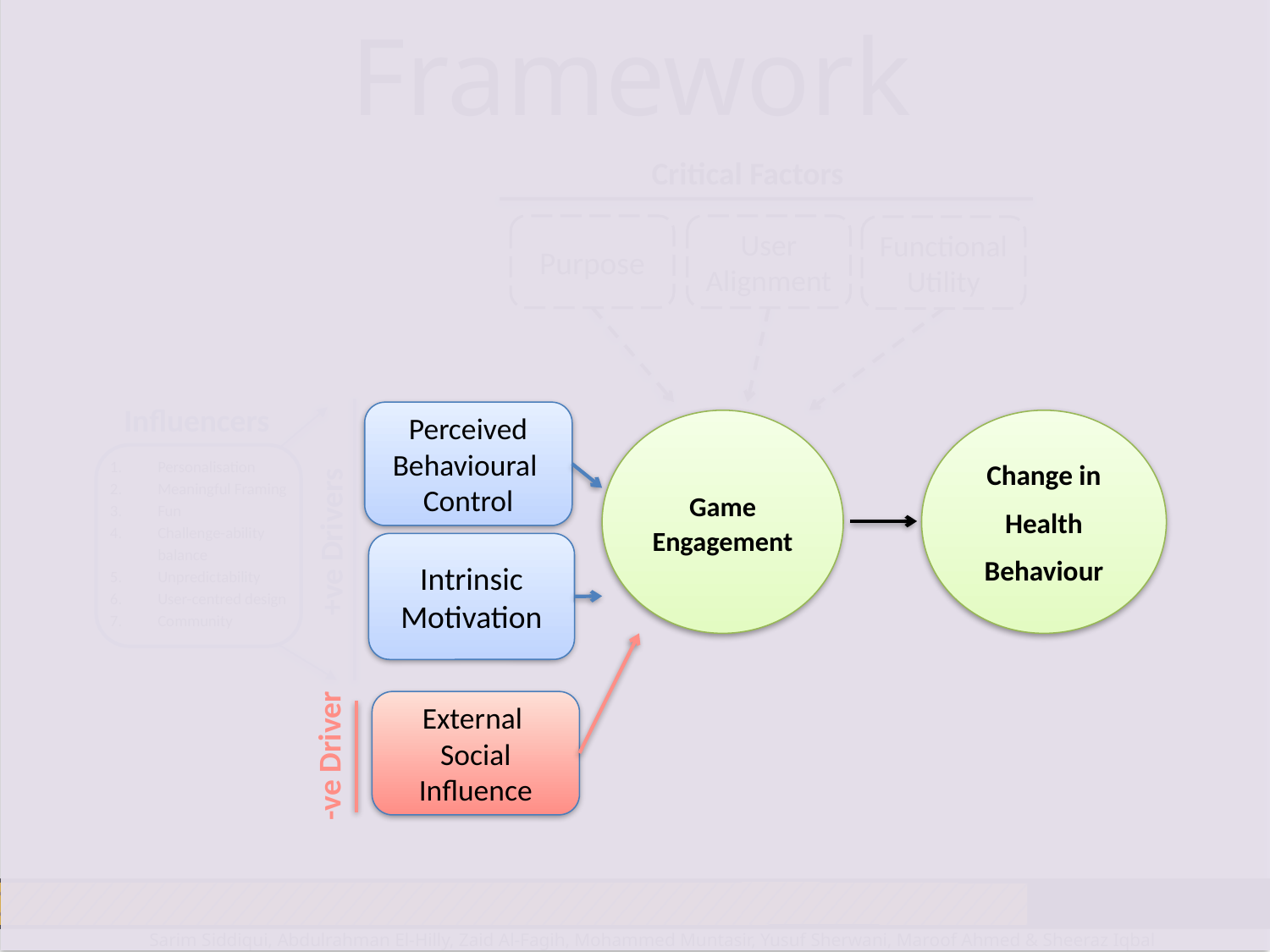

Framework
Critical Factors
Purpose
User Alignment
Functional
Utility
Influencers
Perceived
Behavioural
Control
Change in
Health
Behaviour
Game Engagement
Personalisation
Meaningful Framing
Fun
Challenge-ability balance
Unpredictability
User-centred design
Community
+ve Drivers
Intrinsic
Motivation
External
Social
Influence
-ve Driver
Sarim Siddiqui, Abdulrahman El-Hilly, Zaid Al-Fagih, Mohammed Muntasir, Yusuf Sherwani, Maroof Ahmed & Sheeraz Iqbal

## Slide 16
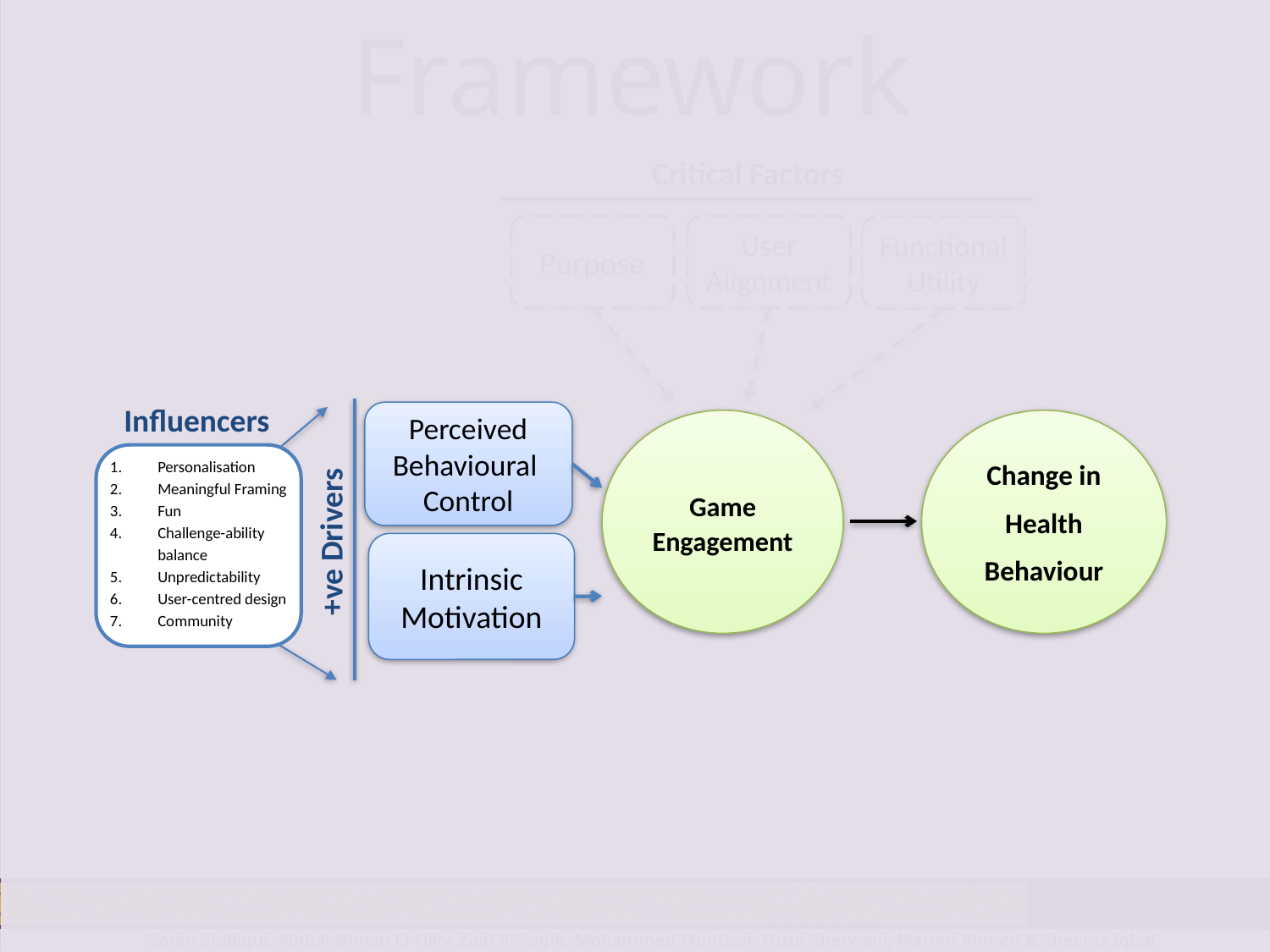

Framework
Critical Factors
Purpose
User Alignment
Functional
Utility
Influencers
Perceived
Behavioural
Control
Change in
Health
Behaviour
Game Engagement
Personalisation
Meaningful Framing
Fun
Challenge-ability balance
Unpredictability
User-centred design
Community
+ve Drivers
Intrinsic
Motivation
Sarim Siddiqui, Abdulrahman El-Hilly, Zaid Al-Fagih, Mohammed Muntasir, Yusuf Sherwani, Maroof Ahmed & Sheeraz Iqbal

## Slide 17
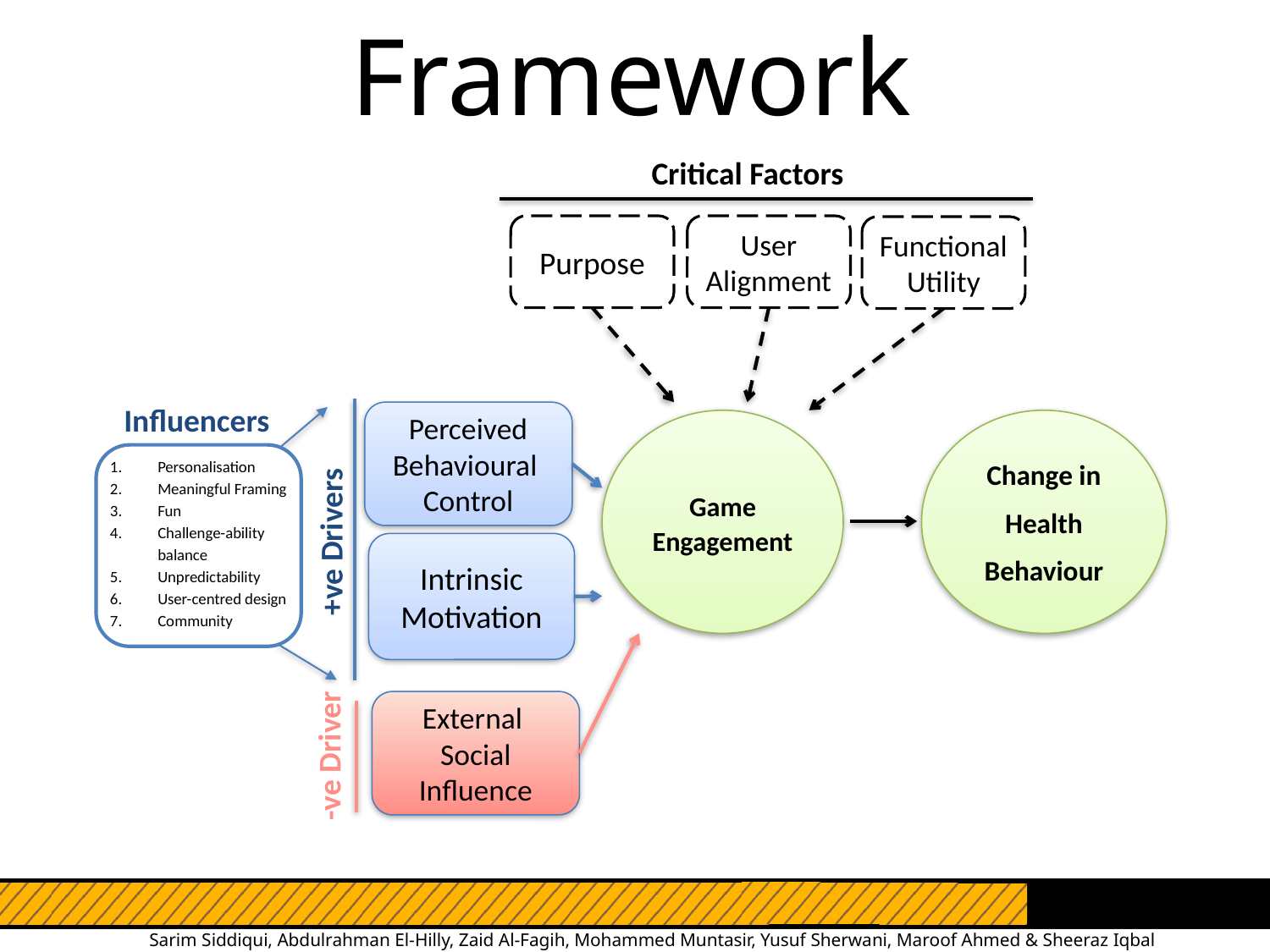

Framework
Critical Factors
Purpose
User Alignment
Functional
Utility
Influencers
Perceived
Behavioural
Control
Change in
Health
Behaviour
Game Engagement
Personalisation
Meaningful Framing
Fun
Challenge-ability balance
Unpredictability
User-centred design
Community
+ve Drivers
Intrinsic
Motivation
External
Social
Influence
-ve Driver
Sarim Siddiqui, Abdulrahman El-Hilly, Zaid Al-Fagih, Mohammed Muntasir, Yusuf Sherwani, Maroof Ahmed & Sheeraz Iqbal

## Slide 18
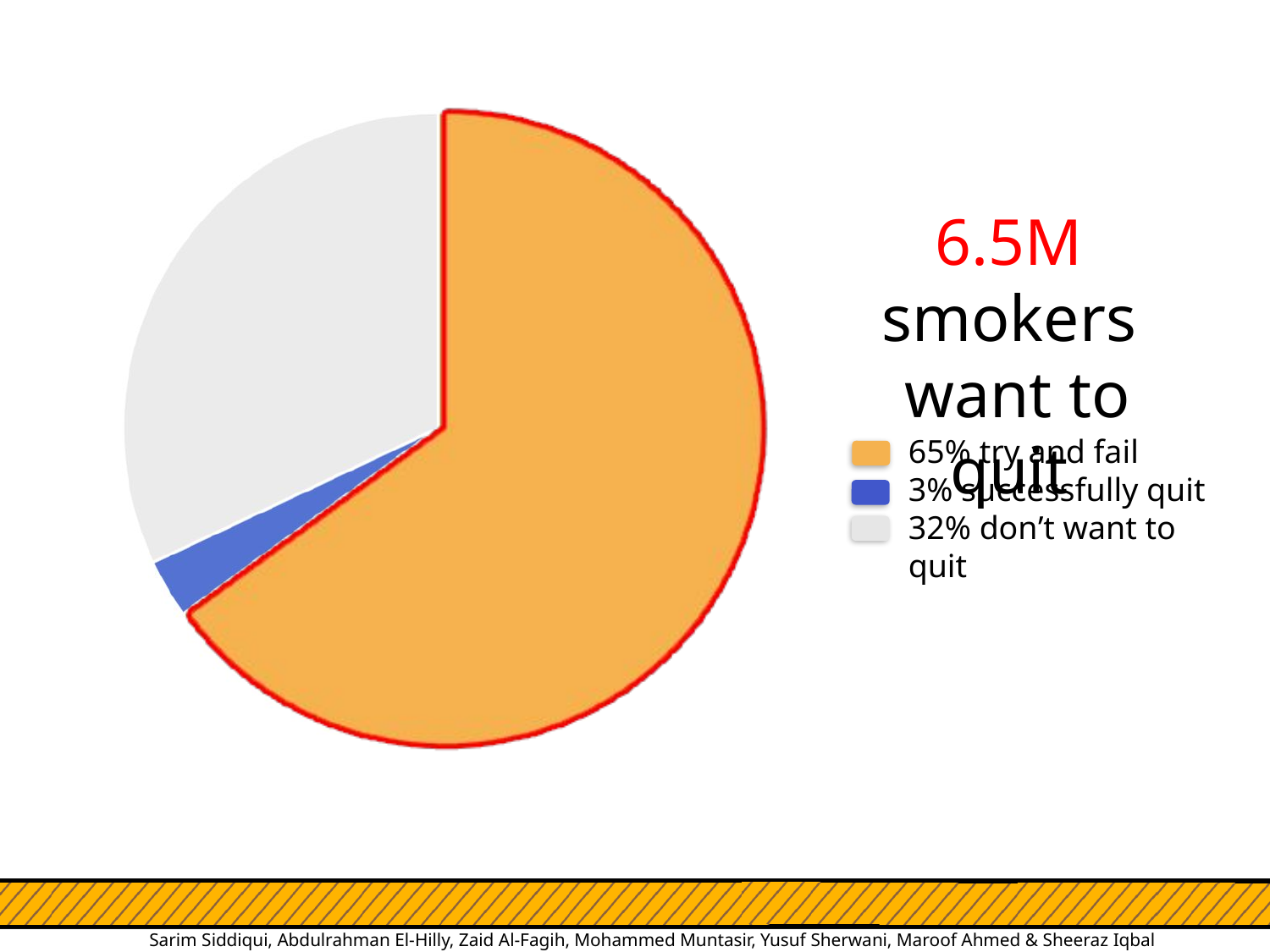

6.5M smokers
 want to quit
65% try and fail
3% successfully quit
32% don’t want to quit
Sarim Siddiqui, Abdulrahman El-Hilly, Zaid Al-Fagih, Mohammed Muntasir, Yusuf Sherwani, Maroof Ahmed & Sheeraz Iqbal

## Slide 19
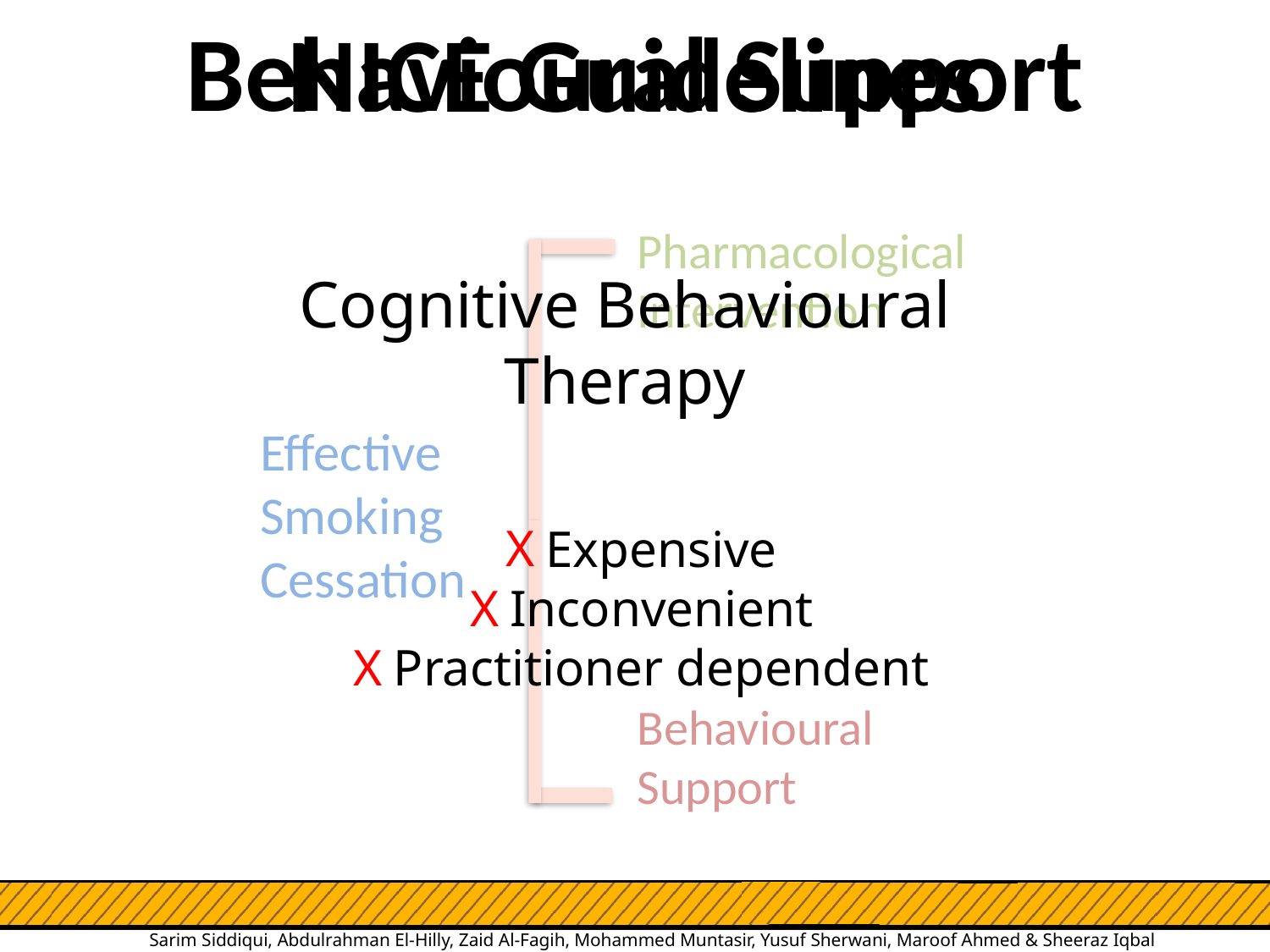

NICE Guidelines
Behavioural Support
Pharmacological
Intervention
Cognitive Behavioural Therapy
Effective
Smoking
Cessation
Expensive
Inconvenient
Practitioner dependent
Behavioural Support
Sarim Siddiqui, Abdulrahman El-Hilly, Zaid Al-Fagih, Mohammed Muntasir, Yusuf Sherwani, Maroof Ahmed & Sheeraz Iqbal

## Slide 20
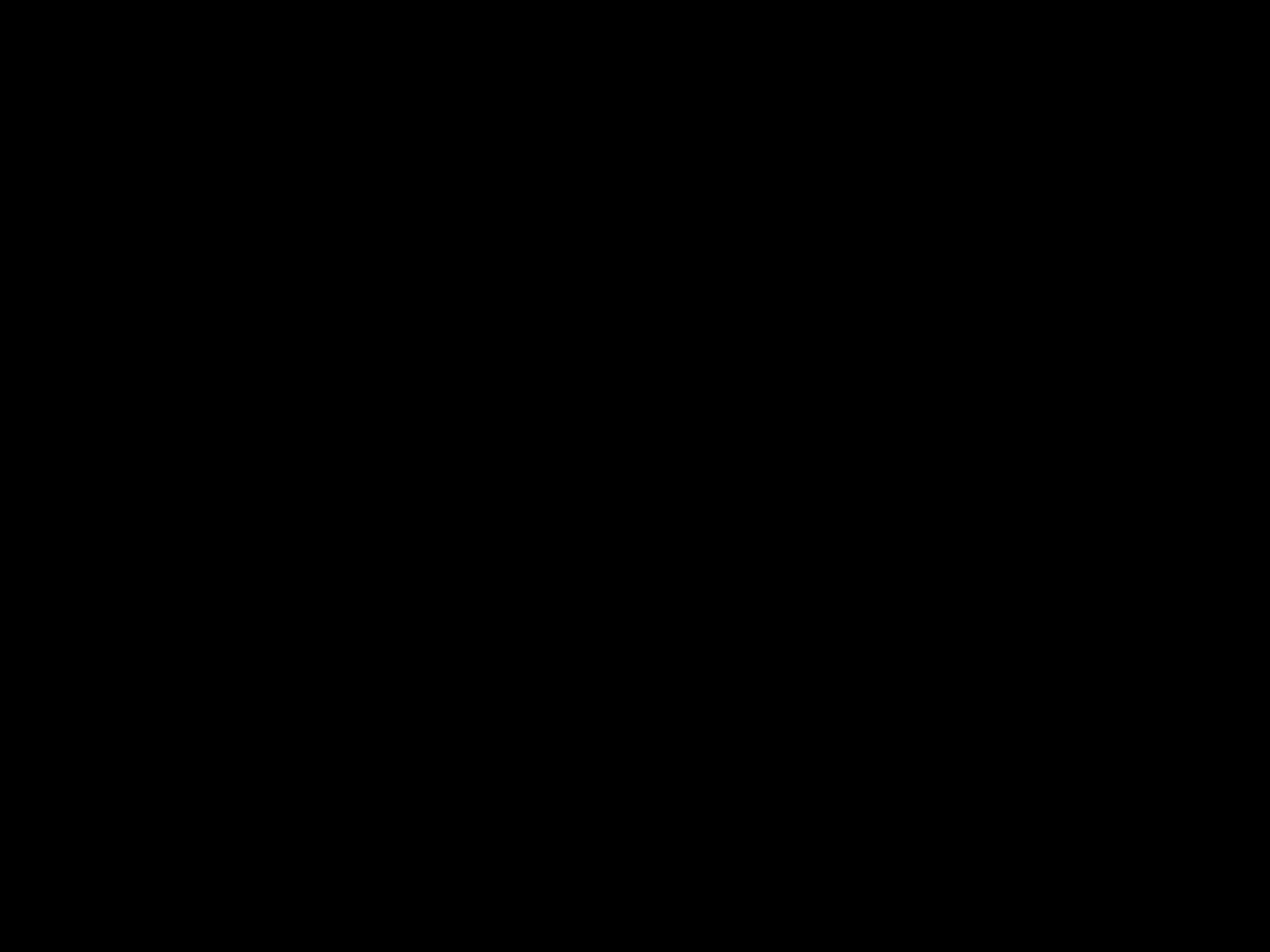

## Slide 21
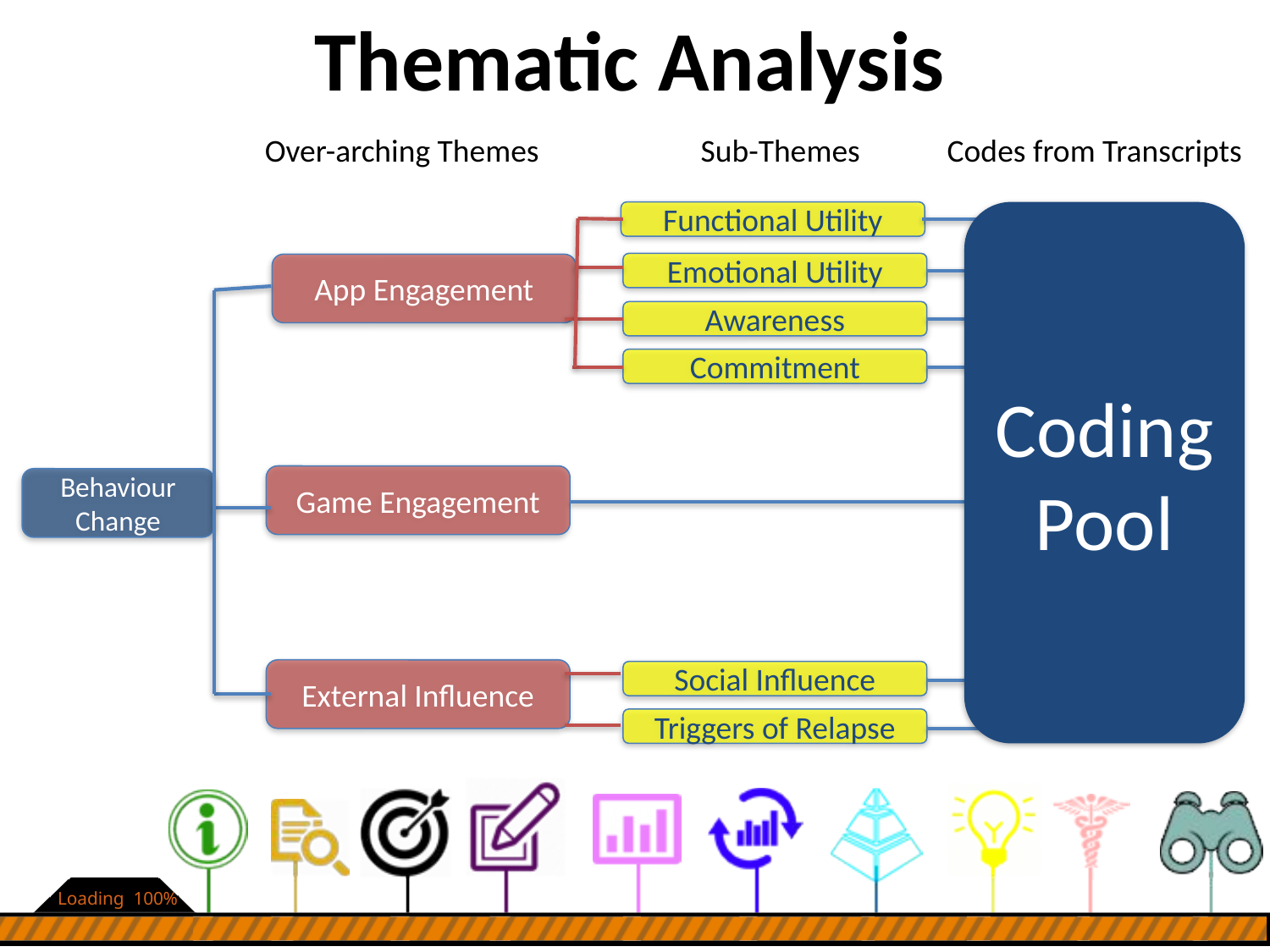

Thematic Analysis
Over-arching Themes
Sub-Themes
Codes from Transcripts
Functional Utility
Coding
Pool
Emotional Utility
App Engagement
Awareness
Commitment
Game Engagement
Behaviour Change
External Influence
Social Influence
33 Y/O Female: “I didn’t care much at the beginning but once I was on level 9 I did not want to go down so tired my best to stay at the same level...I didn’t think much about gaining levels but I really did not want to lose levels”
Triggers of Relapse
Loading 100%

## Slide 22
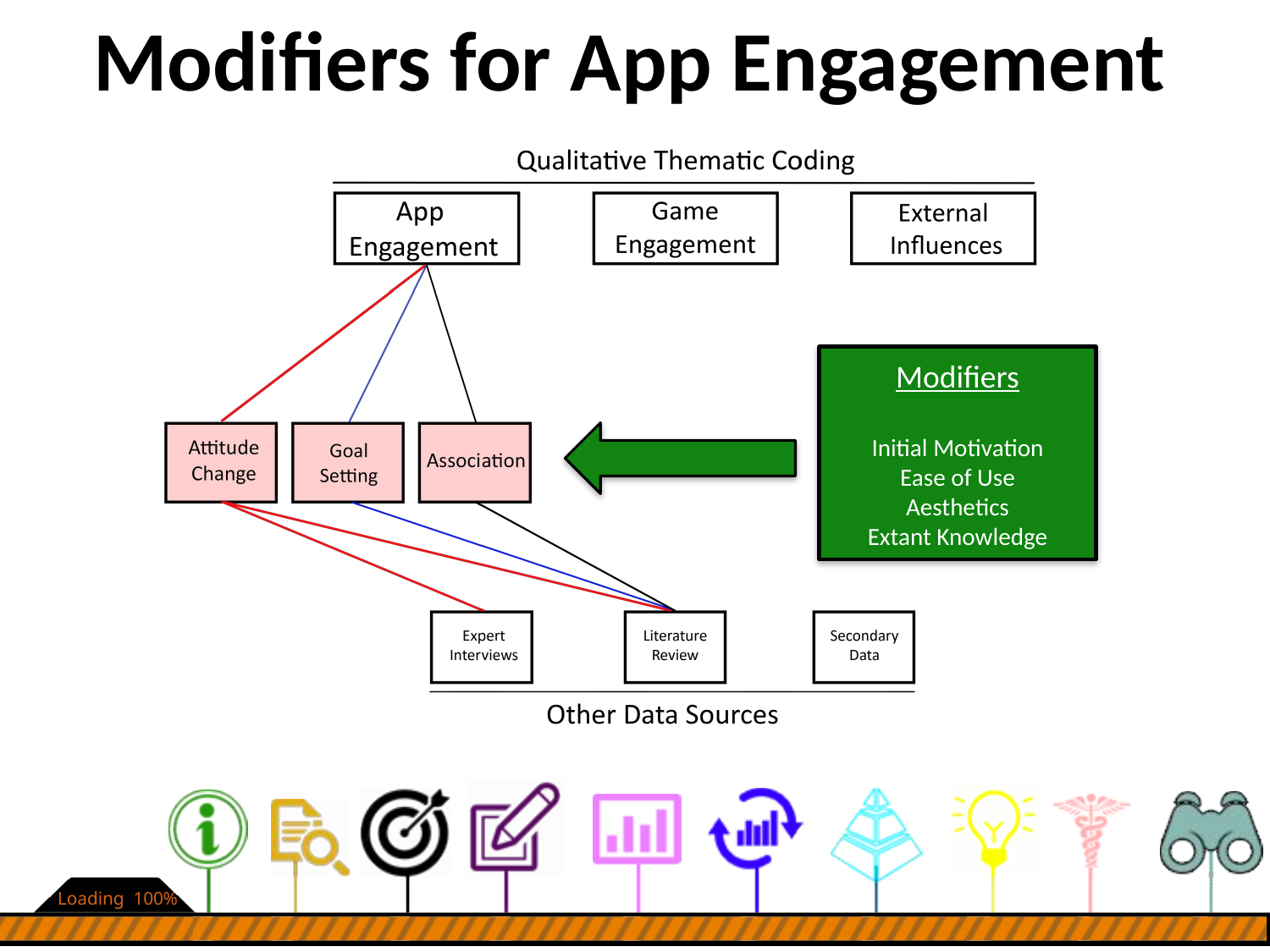

Modifiers for App Engagement
Modifiers
Initial Motivation
Ease of Use
Aesthetics
Extant Knowledge
33 Y/O Female: “I didn’t care much at the beginning but once I was on level 9 I did not want to go down so tired my best to stay at the same level...I didn’t think much about gaining levels but I really did not want to lose levels”
Loading 100%

## Slide 23
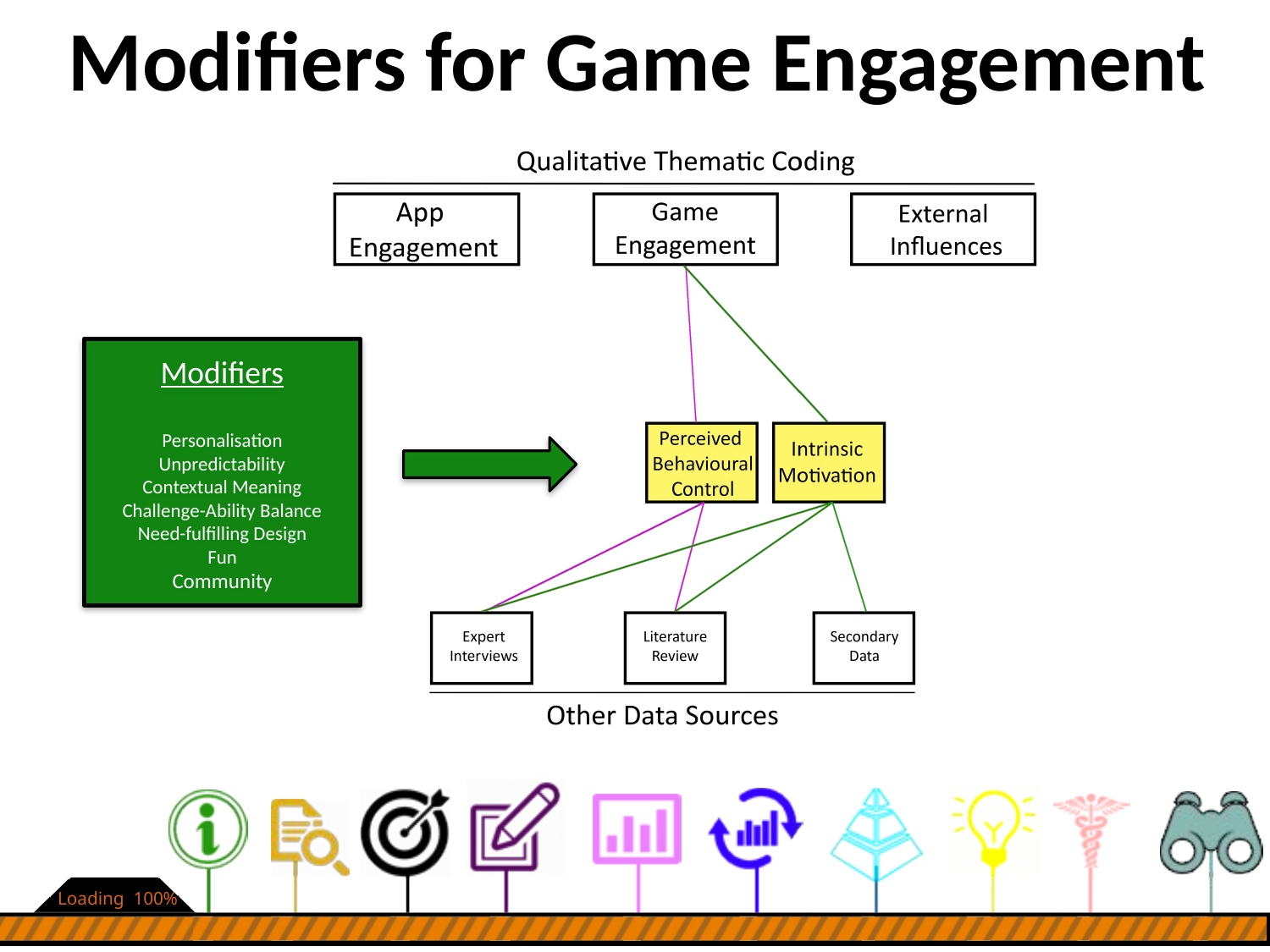

Modifiers for Game Engagement
Modifiers
Personalisation
Unpredictability
Contextual Meaning
Challenge-Ability Balance
Need-fulfilling Design
Fun
Community
33 Y/O Female: “I didn’t care much at the beginning but once I was on level 9 I did not want to go down so tired my best to stay at the same level...I didn’t think much about gaining levels but I really did not want to lose levels”
Loading 100%

## Slide 24
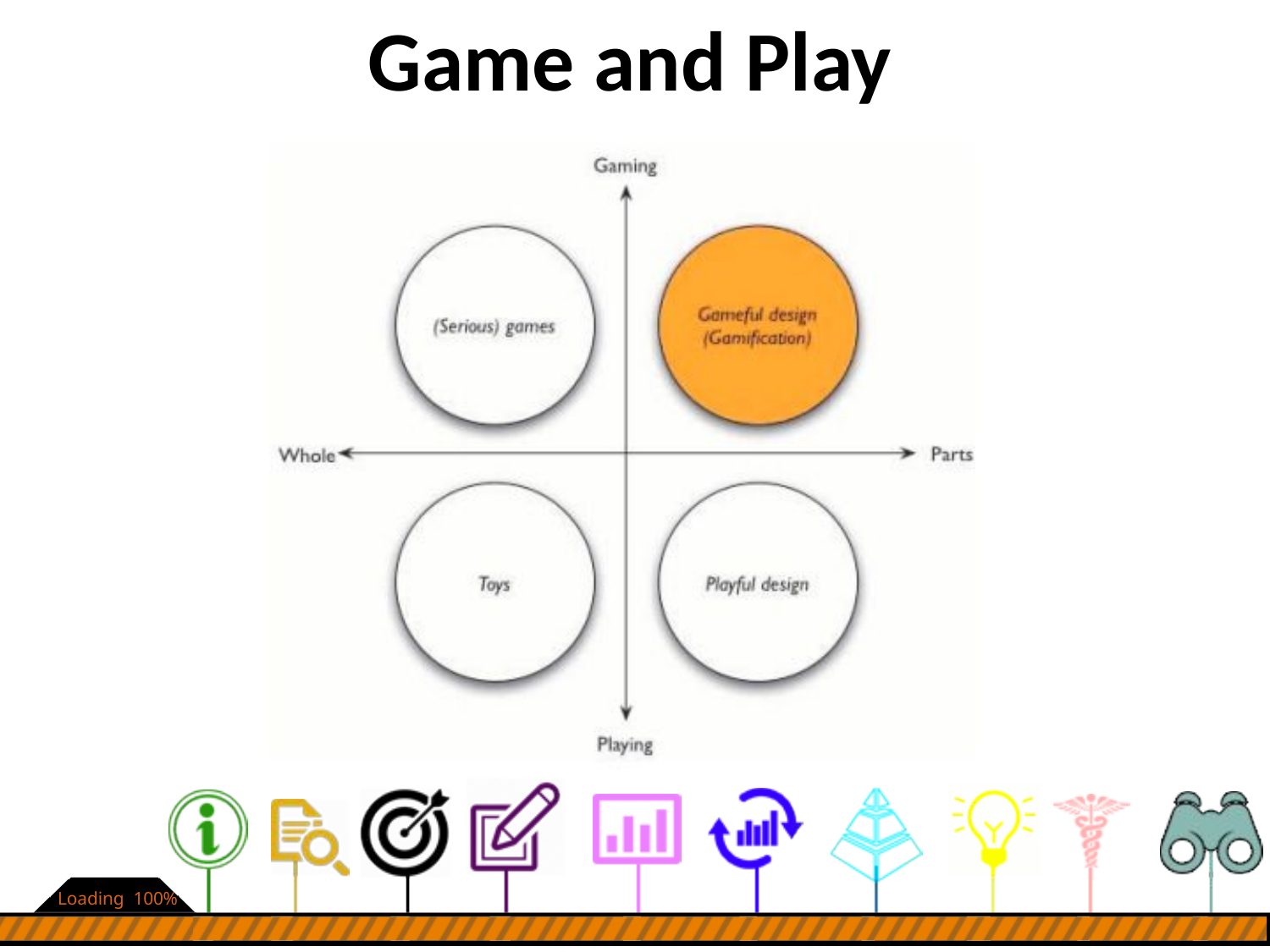

Game and Play
33 Y/O Female: “I didn’t care much at the beginning but once I was on level 9 I did not want to go down so tired my best to stay at the same level...I didn’t think much about gaining levels but I really did not want to lose levels”
Loading 100%

## Slide 25
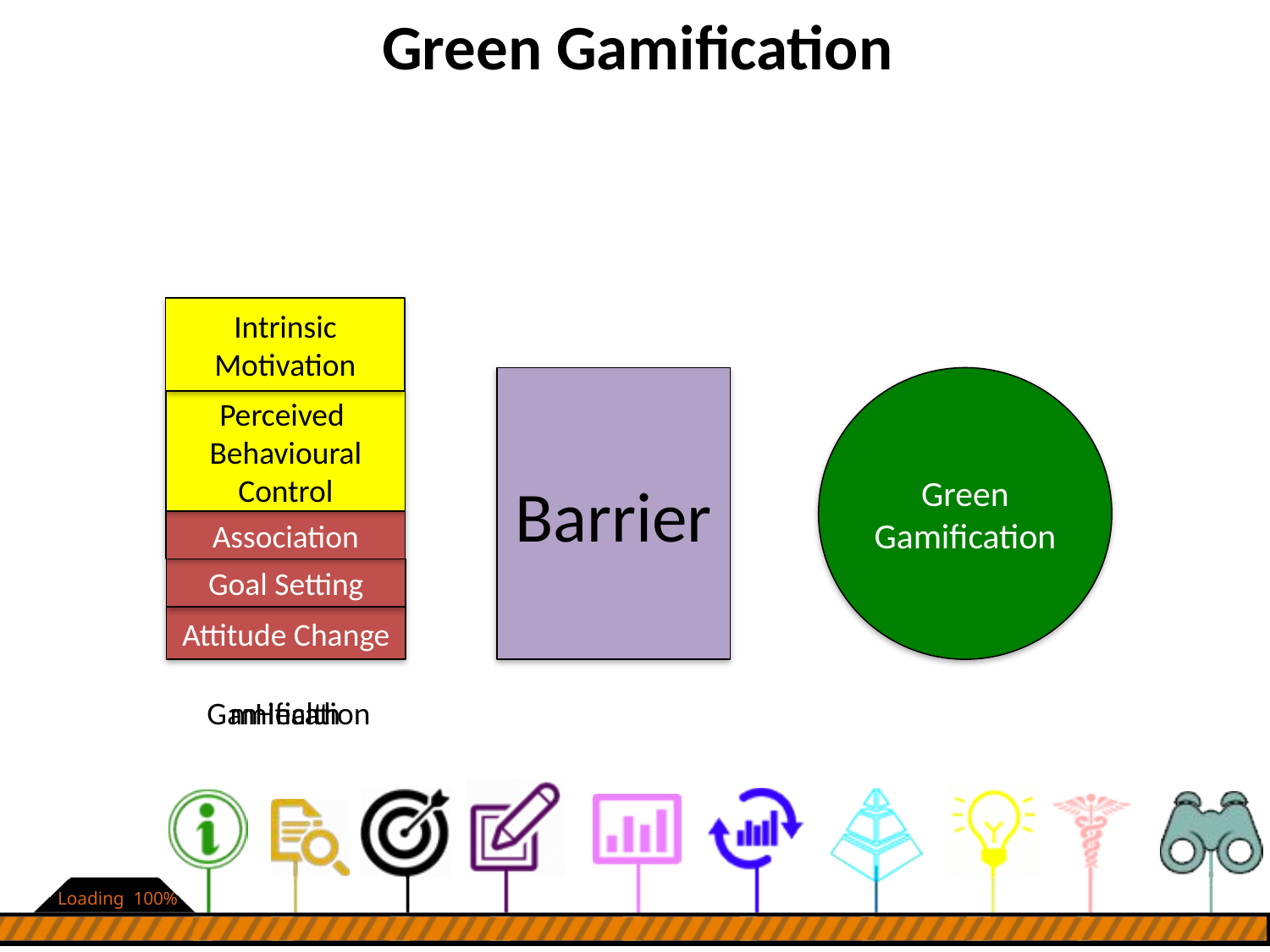

Green Gamification
Intrinsic
Motivation
Barrier
Green
Gamification
Perceived
Behavioural
Control
Association
Goal Setting
Attitude Change
Gamification
mHealth
Loading 100%

## Slide 26
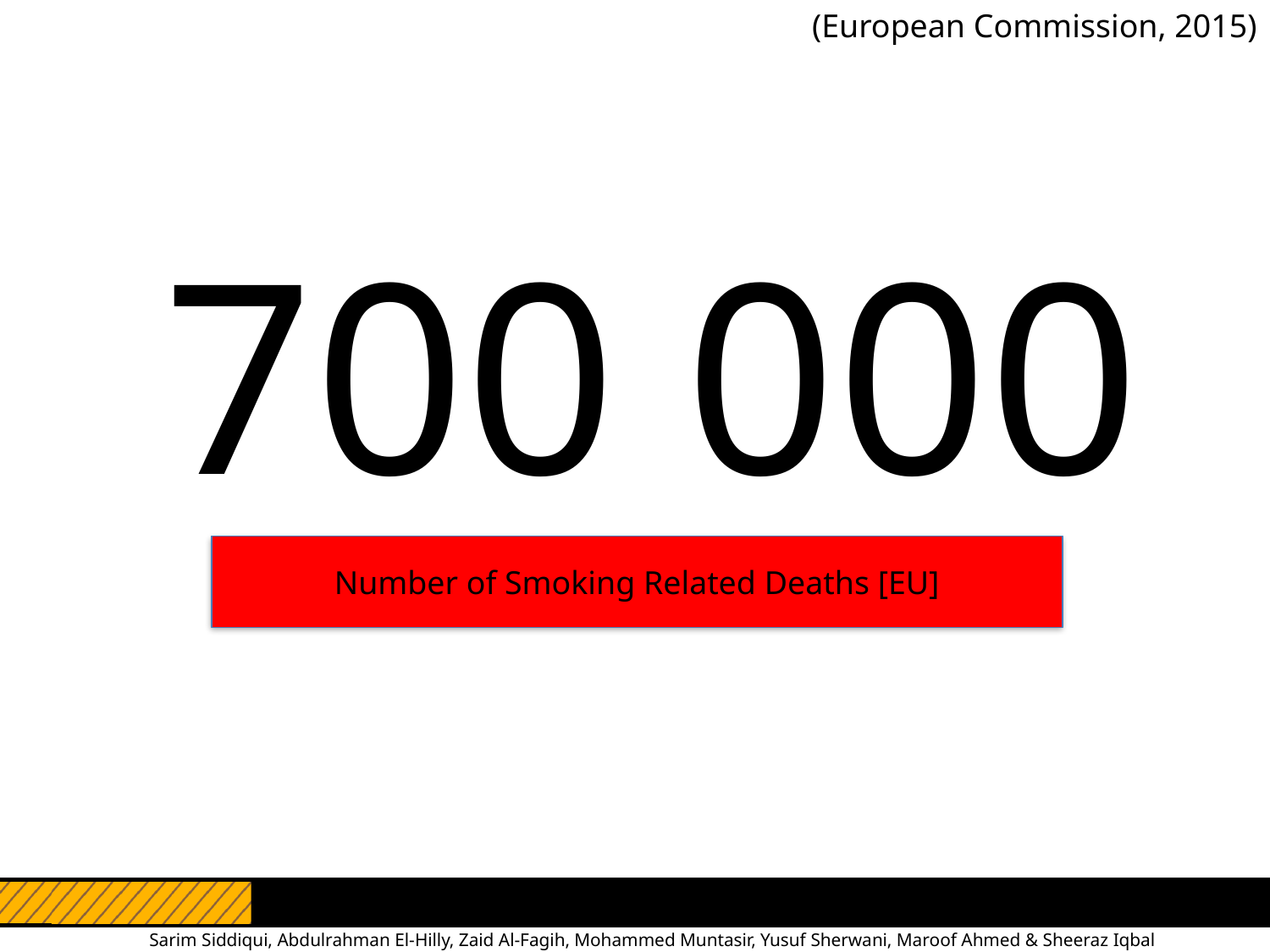

(European Commission, 2015)
700 000
Number of Smoking Related Deaths [EU]
Sarim Siddiqui, Abdulrahman El-Hilly, Zaid Al-Fagih, Mohammed Muntasir, Yusuf Sherwani, Maroof Ahmed & Sheeraz Iqbal

## Slide 27
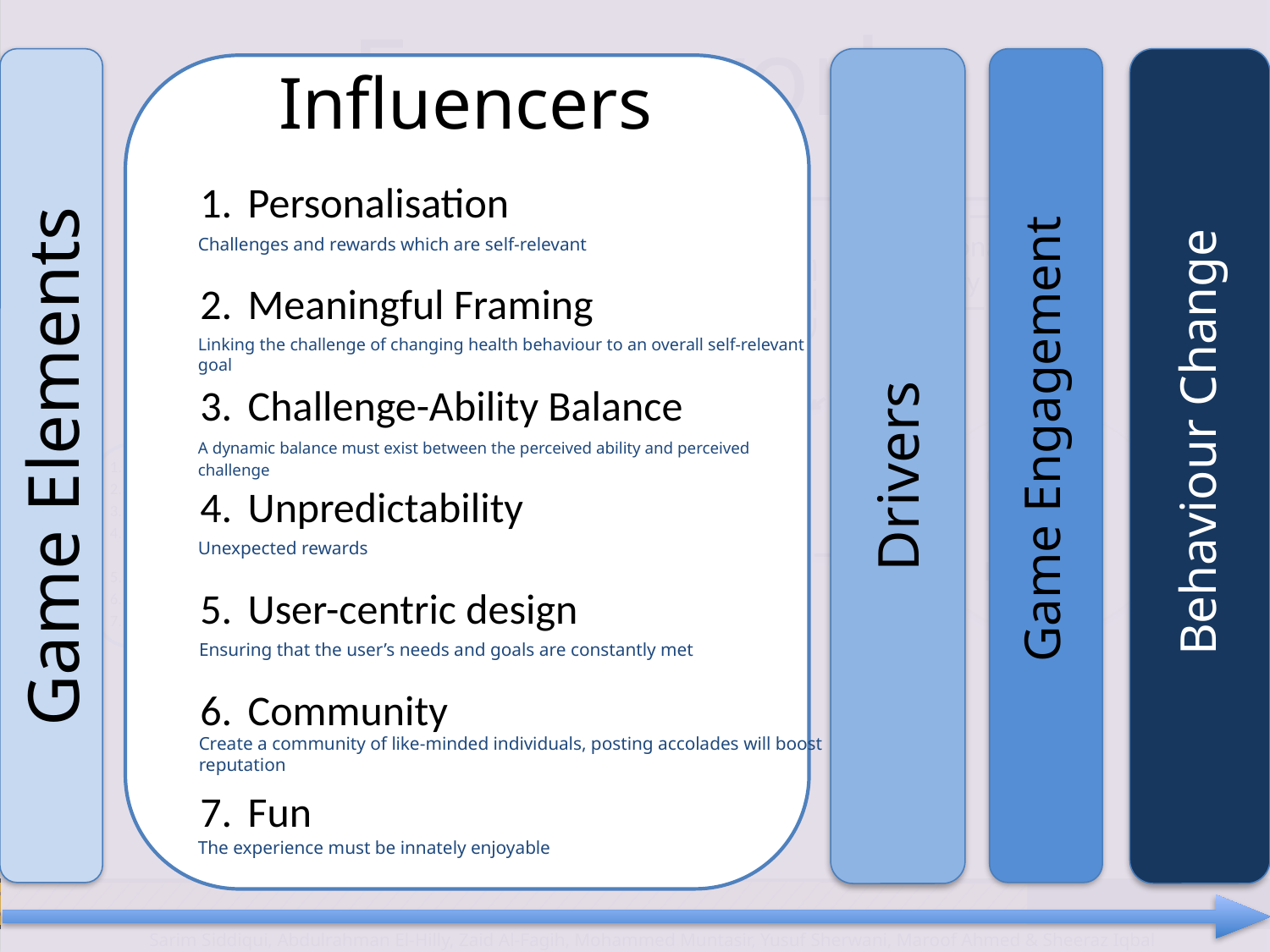

Framework
Influencers
Personalisation
Meaningful Framing
Challenge-Ability Balance
Unpredictability
User-centric design
Community
Fun
Critical Factors
Functional
Utility
Challenges and rewards which are self-relevant
Purpose
User Alignment
Linking the challenge of changing health behaviour to an overall self-relevant goal
Influencers
Game Engagement
Behaviour Change
Change in
Health
Behaviour
Game Elements
A dynamic balance must exist between the perceived ability and perceived challenge
Drivers
Perceived
Behavioural
Control
Game Engagement
Personalisation
Meaningful Framing
Fun
Challenge-ability balance
Unpredictability
User-centred design
Community
Unexpected rewards
+ve Drivers
Intrinsic
Motivation
Ensuring that the user’s needs and goals are constantly met
Create a community of like-minded individuals, posting accolades will boost
reputation
The experience must be innately enjoyable
Sarim Siddiqui, Abdulrahman El-Hilly, Zaid Al-Fagih, Mohammed Muntasir, Yusuf Sherwani, Maroof Ahmed & Sheeraz Iqbal
